# Two conformations of DNA polymerase D-PCNA-DNA, an archaeal replisome complex, revealed by cryo-electron microscopy

**DOI:** 10.1186/s12915-020-00889-y

**Published:** 2020-10-28

**Authors:** Kouta Mayanagi, Keisuke Oki, Naoyuki Miyazaki, Sonoko Ishino, Takeshi Yamagami, Kosuke Morikawa, Kenji Iwasaki, Daisuke Kohda, Tsuyoshi Shirai, Yoshizumi Ishino

**Affiliations:** 1 Medical Institute of Bioregulation, Kyushu University, 3-1-1 Maidashi, Higashi-ku, Fukuoka-shi, Fukuoka, 812-8582 Japan; 2 Department of Bioscience and Biotechnology, Graduate School of Bioresource and Bioenvironmental Sciences, Kyushu University, 744 Motooka, Nishi-ku, Fukuoka, Fukuoka 819-0395 Japan; 3 Institute for Protein Research, Osaka University, 3-2 Yamadaoka, Suita, Osaka, 565-0871 Japan; 4 Present address: Life Science Center for Survival Dynamics Tsukuba Advanced Research Alliance (TARA), University of Tsukuba, 1-1-1 Tennodai, Tsukuba, Ibaraki, 305-8577 Japan; 5 Department of Gene Mechanisms, Graduate School of Biostudies, Kyoto University, Yoshida-konoemachi, Sakyo-ku, Kyoto, 606-8501 Japan; 6 Department of Bioscience, Nagahama Institute of Bio-Science and Technology, Tamura 1266, Nagahama, Shiga 526-0829 Japan

**Keywords:** Archaea, Replisome, PolD, PCNA, Processive DNA synthesis

## Abstract

**Background:**

DNA polymerase D (PolD) is the representative member of the D family of DNA polymerases. It is an archaea-specific DNA polymerase required for replication and unrelated to other known DNA polymerases. PolD consists of a heterodimer of two subunits, DP1 and DP2, which contain catalytic sites for 3′-5′ editing exonuclease and DNA polymerase activities, respectively, with both proteins being mutually required for the full activities of each enzyme. However, the processivity of the replicase holoenzyme has additionally been shown to be enhanced by the clamp molecule proliferating cell nuclear antigen (PCNA), making it crucial to elucidate the interaction between PolD and PCNA on a structural level for a full understanding of its functional relevance. We present here the 3D structure of a PolD-PCNA-DNA complex from *Thermococcus kodakarensis* using single-particle cryo-electron microscopy (EM).

**Results:**

Two distinct forms of the PolD-PCNA-DNA complex were identified by 3D classification analysis. Fitting the reported crystal structures of truncated forms of DP1 and DP2 from *Pyrococcus abyssi* onto our EM map showed the 3D atomic structural model of PolD-PCNA-DNA. In addition to the canonical interaction between PCNA and PolD via PIP (PCNA-interacting protein)-box motif, we found a new contact point consisting of a glutamate residue at position 171 in a β-hairpin of PCNA, which mediates interactions with DP1 and DP2. The DNA synthesis activity of a mutant PolD with disruption of the E171-mediated PCNA interaction was not stimulated by PCNA in vitro.

**Conclusions:**

Based on our analyses, we propose that glutamate residues at position 171 in each subunit of the PCNA homotrimer ring can function as hooks to lock PolD conformation on PCNA for conversion of its activity. This hook function of the clamp molecule may be conserved in the three domains of life.

**Supplementary information:**

The online version contains supplementary material available at 10.1186/s12915-020-00889-y.

## Background

Deoxyribonucleic acid (DNA) replication is a fundamental process that is essential in all three domains of life: Archaea, Bacteria, and Eukarya. The replisome is a large protein complex that processes DNA replication, and DNA polymerase (DNAP) plays a central role in the synthesis of nascent DNA strands. To date, DNAPs have been classified into seven families: A, B, C, D, E, X, and Y, according to the amino acid sequence conservation [1–4]. Among the archaeal DNAPs, DNA polymerase Bs (PolBs), especially those found in hyperthermophilic archaea, such as *Pyrococcus furiosus* PolB (PfuPolB) [5] or *Thermococcus kodakarensis* PolB (TkoPolB) [6], have been structurally and biochemically studied in detail and are widely applied in biotechnology for PCR because of their extreme thermostability and outstanding fidelity [7]. The distinct DNA polymerase D (PolD), originally discovered in *Pyrococcus furiosus* as its second DNA polymerase (PolII) after PfuPolB (PolI) [8], has been subsequently identified in most of the archaeal organisms except for the members of the phylum Crenarchaeota [9]. The PolD holoenzyme consists of two subunits, DP1 and DP2, which contain the catalytic sites of the 3′–5′ exonuclease and the 5′–3′ DNA polymerase, respectively.

Originally, family B-DNA polymerase has been thought to be the replicase in Archaea because the growth of a haloarchaeon has been shown to be suppressed by aphidicolin, an inhibitor for eukaryotic Polα-like DNA polymerases [10]. The function of PolB as a replicase in Archaea appeared to be further supported by its strong 3′–5′ exonuclease in vitro. On the other hand, the 3′–5′ exonuclease of PolD is comparable to that of PolB. In addition, PolD obviously prefers the reaction for strand extension over gap-filling in vitro [11]. Therefore, it has been suspected that PolB and PolD are both involved in replication, being involved in the leading strand and the lagging strand synthesis, respectively [12, 13]. A genetic study in *Halobacterium* indicated that both the *polB* and *dp1*, *dp2 * (for PolD) genes are essential for its viability, also supporting the hypothesis of two replicative polymerases [14]. However, more recent studies have shown that the *polB* gene can be deleted without any growth defect in the two euryarchaea, *Methanococcus maripaludis* [15] and *T*. *kodakarensis* [16]. These findings strongly suggest that PolD, rather than PolB, is the essential genome replicating enzyme in many, if not all, Archaea (except for Crenarchaeota) and stimulates the interest in detailed characterization of PolD.

Although PolD has been discovered in *P*. *furiosus* in 1997 [8], the 3D structure of this enzyme has not been solved for many years, due to its low stability. However, individual crystal structures of the core domains of DP1 and DP2 from *P*. *abyssi* have been reported in 2016 [17]. The DP1 subunit belongs to the calcineurin-like phosphodiesterase superfamily [18, 19], and the reported crystal structure showed the highest similarity to the Mre11 exo-/endonuclease [17]. By contrast, DP2 has a distinct structure, with no sequence similarity to other known DNA polymerases. However, structural analysis has revealed an unexpected homology between the core catalytic domain of DP2 and the double-psi β-barrel (DPBB) core domain of the DNA-directed RNA polymerases (RNAP) that are involved in transcription in all three domains of life and many large viruses [17]. Our recent biochemical analysis of *T*. *kodakarensis* PolD by electron microscopy (EM) visualized the precise subunit composition and arrangement for the first time [20], and most recently, the 3D complex structure of *P*. *abyssi* DP1-DP2 complex with DNA resolved by cryo-electron microscopy (cryo-EM) has been reported by others [21].

The processivity of replicase is generally enhanced by the clamp molecule, such as the proliferating cell nuclear antigen (PCNA) in Archaea/Eukaryote and the PolIII β subunit in Bacteria. For example, DNA synthesis by *P*. *furiosus* and *T*. *kodakarensis* PolDs is enhanced by its cognate PCNAs in vitro [22, 23]. Therefore, to understand the structure and functions of the archaeal replisome, it is crucial to elucidate the interaction between PolD and PCNA. We have performed single-particle analysis of various archaeal complexes containing PCNA, such as replication factor C (RFC)-PCNA-DNA [24], DNA ligase (Lig)-PCNA-DNA [25], PolB-PCNA-DNA [26, 27], FEN1-PCNA-DNA [28], and FEN1-Lig-PCNA-DNA [28]. These structural analyses led to the proposal that PCNA serves as the platform on which various factors are assembled to perform their specific roles in DNA replication via sequential reactions. These analyses have also shown that PCNA is involved not only in tethering these factors around DNA but also in the regulation of their activities.

Here, we present a direct view of the 3D structure of the archaeal replisome containing PCNA-bound *T*. *kodakarensis* PolD, which was complexed with a synthetic primed DNA (PolD-DNA-PCNA), by single-particle analysis using cryo-EM. Two distinct structures of a PolD-DNA-PCNA complex, which represent different functional modes, were observed. We also successfully visualized flexible loop regions which appeared disordered in the previous studies. These loop regions are used for previously unnoticed contacts between PolD and PCNA, in addition to the well-known PIP (PCNA-interacting protein)-box mediated interaction. These newly identified interactions are mediated by E171 of PCNA and may work as hooks to lock the conformation of PolD in either the synthesis or the editing modes on PCNA. The direct structural view of the PolD-PCNA complex is essential to fully understand the mechanism of DNA replication in Archaea at the molecular level.

## Results

### Reconstitution of the PolD-PCNA-DNA complex

We reconstituted and purified the PolD-PCNA-DNA complex using DNAs listed in Additional file: Figure S1 by gel filtration chromatography (Additional file: Figure S2A-D). We first used a synthetic primed DNA made of temp45Msss and pri30EMsss (Additional file: Figure S1). The 3′-terminal regions of the DNA were phosphorothioated to protect from 3′-5′ exonuclease of PolD. Furthermore, the proteins and DNA were dissolved in a buffer without magnesium ion. The complex (referred to as PolD-PCNA-DNA (30/45) complex) was successfully purified by gel filtration as a single peak (Additional file: Figure S2A), which contained all the protein components (Additional file: Figure S2B). The absorbance ratios at 260 and 280 nm (A_260_/_280_) of the peak fraction indicated that this fraction also contained the DNA substrate. This isolated complex was subjected to EM single-particle analysis.

### Single-particle image analysis

Representative electron microscopic images from the peak fraction are shown in Additional file: Figure S3A, B and C (S3D is for the complex containing a primed DNA (25/35), described below). The cryo-EM images showed that the complex tends to form tetrameric or pentameric clusters (marked in the figures S3B, C). Such clusters were not observed in the negatively stained images prepared by five-fold dilution of the protein concentration (Additional file: Figure S3A); thus, the interaction between the complex particles seems to be weak. The 3D classification analysis of the complex images revealed that at least two stable forms of complex coexist in solution (Additional file: Figure S4: Form A and Form B). A substantial difference in the orientations of PolD relative to PCNA was observed between the two forms: PCNA in Form A (see class 8 in Additional file: Figure S4) horizontally overlapped with PolD whereas in Form B, a notable tilt was observed for PolD (see class 1 in Additional file: Figure S4).

**Fig. 1:**
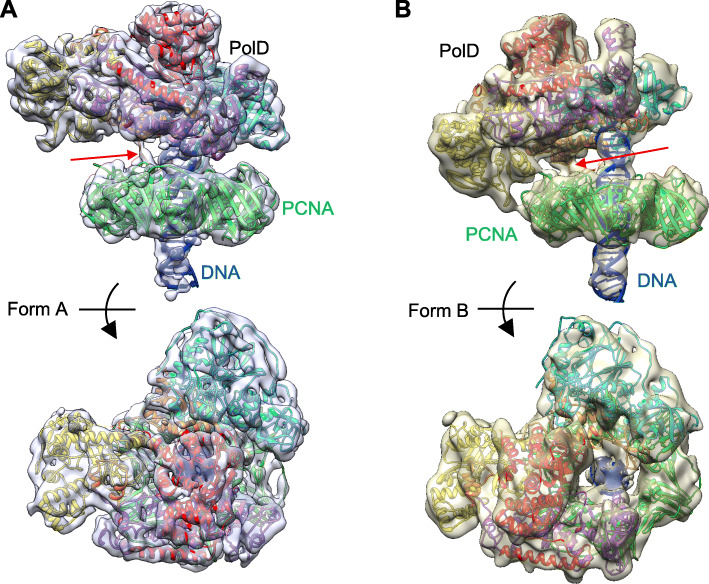
The 3D EM maps of PolD-PCNA-DNA complex. **a** Side view (upper) and top view (lower) of Form A. The crystal structures of PCNA and DP1 are shown in green and cyan ribbon models. The N-terminal, catalytic, center, and C-terminal domains of DP2 are shown in red, purple, yellow, and orange ribbon models, respectively. **b** Side view (upper) and top view (lower) of Form B. The ribbon models are colored as in **a**. The PCNA interacting with C-terminal loop of DP2, and harboring the PIP-box, are indicated by a red arrow in **a** and **b**

Next, we tried to improve the resolution, by more strictly refining conditions for sample preparation and EM observation. We successfully obtained a higher resolution map (6.9 Å) as shown in Fig. 1a (see also Additional file: Figures S5 and S6) of the Form A complex, consisting of PolD, PCNA, and primed DNA (25/35) (Additional file: Figure S1; pri25EMssss and temp35EMssss). The ternary complex was isolated as in the case of the primed DNA (30/45) (Additional file: Figures S2C and D). The procedures for the 3D structure analysis are described in more detail in the Materials and Methods section (see also Additional file: Figures S3D, S5 and S6). While the ratios of complex particles forming clusters were reduced, the ratio of free isolated complex particles was increased. Notably, in our 3D classification analysis, we could not detect a 3D map corresponding to Form B from the complex using the shorter DNA (Additional file: Figure S5). This result suggests that Form A, but not Form B, was selectively stabilized in this sample preparation, thus leading to the improvement of the resolution.

### Overall structure of the PolD-PCNA-DNA complex

The 3D EM maps of A and B forms of the PolD-PCNA-DNA complexes are shown in Fig. 1. Both maps revealed a two-layered structure, which consisted of the bottom hexagonal PCNA ring and PolD on top of it. A rod-shaped density, passing through the PCNA channel almost perpendicularly and extending to PolD in both maps, appears to represent the double-stranded DNA.

**Fig. 2:**
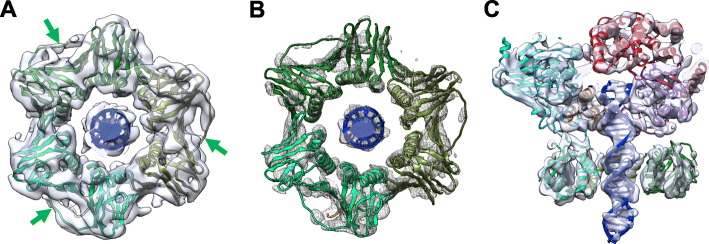
Docking of the TkoPCNA crystal structures on the 3D map of Form A. **a** The three subunits of the TkoPCNA are colored in different green. The interdomain connecting loops (IDCLs) are indicated by green arrows. **b** The same Form A map, in different threshold representation from **a**, to show the fit of alpha helices of PCNA. **c** Cross-sectional side view of Form A map. An atomic model of 25 bp B-DNA (blue) is docked into the map. The ribbon models of PolD are colored as in Fig. 1

In the Form A map, which was obtained from the complex using the (25/35) DNA, the rod-shape density is clearly visualized as typical B-form DNA (Fig. 2 and Additional file: Figure S7). We confirmed the complete visualization of the double-stranded region used for complex reconstruction by docking the atomic model of the 25 bp B-DNA stretch onto the map. A similar DNA orientation (i.e., perpendicular to the clamp) has been observed in the cryo-EM structure of the *E*. *coli* replicase complex consisting of PolIII (α, ε)-β clamp-τ-DNA in the synthesis mode [29]. By contrast, in the Form B map obtained from the complex using the (30/45) DNA, the rod-shaped density did not clearly show a double-stranded helical structure due to limited resolution. The length of the rod-shaped density roughly corresponded to a 25 bp B-DNA molecule, which was shorter than the 30 bp DNA used for complex reconstitution (Fig. 1b and also see Additional file: Figure S8). The ssDNA regions were not clearly visualized in either form, suggesting that the density of the flexible ssDNA was smeared out during the averaging process.

### Fitting crystal structures into the EM structure

As shown in Figs. 1 and 2, the crystal structure of *T*. *kodakaraensis* PCNA (PDB: 3LX1) was fitted into the lower hexagonal region as a rigid body. The inter-domain connecting loop (IDCL) was clearly visualized in both maps for Form A and Form B, allowing us to tightly dock the PCNA trimer into the pseudo-six-fold symmetrical map (Fig. 2a). The 12 α-helices lining up the inner wall of the PCNA channel in the Form A map are well resolved from each other and also from the outer β-sheet regions (Fig. 2b). Similar to the previously solved structures of the PCNA-containing complexes, RFC-PCNA-DNA [24] and PolB-PCNA-DNA [27], no direct contact was observed between the DNA and α-helices of the inner wall of the PCNA ring. By contrast, the DNA duplex was inclined in the PCNA channel, making contact with the inner wall, in the structures of the Lig-PCNA-DNA [25] and FEN-PCNA-DNA complexes [28]. A similarly inclined DNA was observed in the editing complex of the *E*. *coli* replicase [30].

It should be noted that our fitting examination was carried out using the crystal structures of the separated DP1 (Val152-Cys619) and DP2 (Pro4-Asp1039) subunits from *P*. *abyssi* [17]. The sequences of the corresponding regions show high similarity between *P*. *abyssi* and *T*. *kodakarensis*, such as 79% and 89% for DP1 and DP2, respectively. Thus, it enabled us to compare these crystal structures with our EM map. The initial atomic models of Form A and Form B complexes as shown in Additional file: Figures S7 and S8, respectively, were obtained by fitting the crystal structures of DP1 and DP2 from *P*. *abyssi* [17] to our EM map, and then the amino acid sequences of DP1 and DP2 were replaced by those from *T*. *kodakarensis* in the final atomic models (Fig. 1). The overall structure of our model was consistent with that from the published structure of *P*. *abyssi* PolD [21]. In addition, the recent cryo-EM structure of *P*. *abyssi* PolD determined the domain organization of DP2 in more detail and proposed a new nomenclature, in which Anchor and KH-like domains in N-terminal domain, a part of Clamp 1 and DPBB-1 with Accessory-1 domains in catalytic domain, BPBB-2 and Accessory domains in Center domain, and a part of Clamp-1 in C-terminal domain, respectively, were designated. We used the basic domain names to show the DP2 structure simpler in this study.

We presented the “duck”-shaped density of *T*. *kodakarensis* PolD in our previous report [20]. The triangular crystal structure of the *P*. *abyssi* DP1 (PDB: 5IHE) was tightly docked into the “head” region of the “duck” (Additional file: Figure S7). All α-helices and the two long loops (indicated by the two arrowheads in Additional file: Figure S7A) were clearly resolved. We also attempted to fit the OB-fold domain into the map independently. However, this domain remained almost in the original space after fitting with Chimera, indicating that DP1 in the complex retains essentially the same conformation as in the crystal structure. Thus, the entire DP1 crystal structure was docked as a rigid body (Additional file: Figure S7). By contrast, the crystal structure of *P*. *abyssi* DP2 (PDB: 5IJL) did not fit well into the “body” area of the PolD density in either Form A or Form B. However, good fits were observed for both forms when we docked each domain structure of DP2 into the “body” area individually as shown in Additional file: Figures S7 and S8. The N-terminal domain (D46-G285) and the catalytic domain (G681-T992) are linked via the N-terminal “self-assembly α-helix” of the former domain in the crystal structure of DP2 [17]. This α-helix and the short β-sheet (P4-A44, indicated as α1 and β1 in [17]) are bound to the catalytic domain at the same position as in the DP2 crystal (PDB: 5IJL), thereby fitting into the map together with the catalytic domain.

### Modeling DP2CTD

**Fig. 3:**
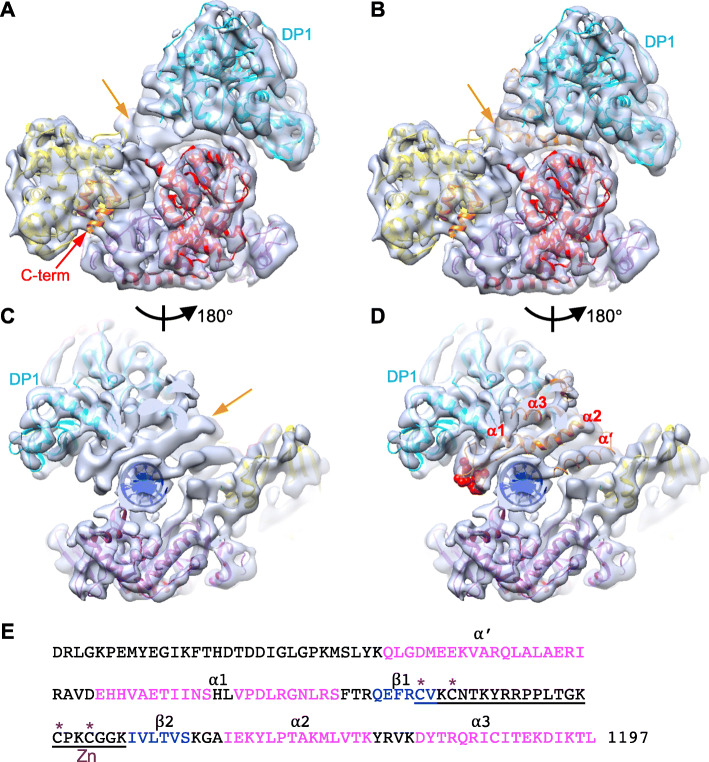
The modeling of the C-terminal domain (CTD) of DP2. **a** Top view of the PolD-PCNA-DNA complex. The crystal structure of DP1 and individual domains of DP2 were fitted as rigid body into the Form A map. The C-terminus of the CTD of DP2 crystal structure is indicated by the red arrow. The orange arrow indicates surplus region where elements corresponding to the crystal structure are not found. **b** Top view of PolD-PCNA-DNA (Form A). The CTD of DP2 was obtained by homology modeling (orange ribbon) and docked into the surplus region. **c** Bottom view (PCNA side) of the PolD-PCNA-DNA. The orange arrow indicates surplus region. **d** Bottom view (PCNA side) of the PolD-PCNA-DNA. The CTD and the Zn finger constructed and fitted by modeling are shown with an orange ribbon and red sphere atomic model, respectively. Slabs in the PCNA region are omitted for clarity for **a** to **d**. **e** Amino acid sequence of DP2 CTD and the predicted secondary structure (magenta: alpha helices, blue: beta sheet). The Zn-binding motif and the four zinc-coordinating cysteines are indicated by underline and asterisks, respectively

**Fig. 4:**
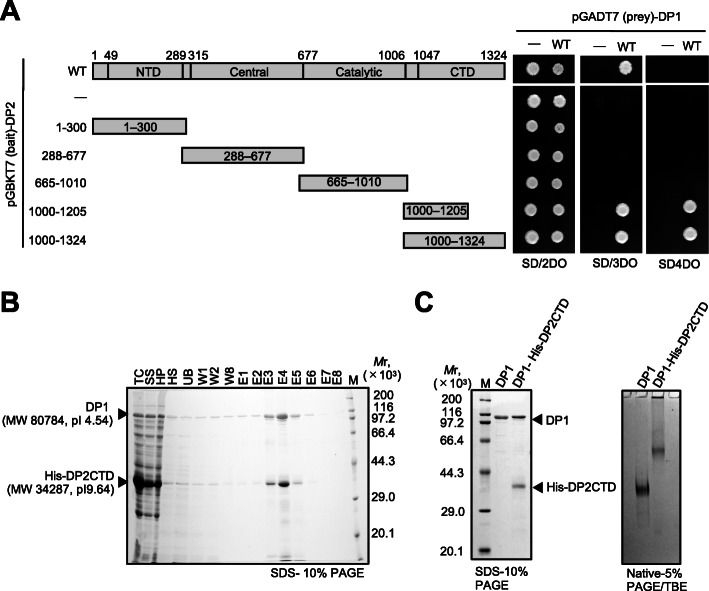
DP1 interacting region of DP2. **a** The interactions between DP1 and DP2 fragments investigated by yeast two-hybrid assay. Domain organization of DP2 is schematically shown. Cell suspensions of each strain were spotted (3 μl of 2 × 10^6^ cell/ml) on S.D. plates without Leu, Trp, and His (middle), and Leu, Trip, His, Ade (right) for two different selection strengths. Transformants on the non-selection plate are shown at the left. (−) indicates the transformants with the bait or prey plasmid without insert DNA. The agar plates were incubated at 30 °C for 4 days, and growing cells were visualized. **b** DP1 and His-tagged DP2CTD were co-purified by TALON metal affinity resin. At each purification step, aliquot was fractionated and subjected to SDS-10% PAGE followed by Coomassie Brilliant Blue staining. Protein size markers were run in lane M and their sizes are indicated on the right side of the gel. TC, total cell; SS, sonication supernatant; HP, heat precipitation; HS, heat supernatant; UB, unbound; W1–8, wash fractions; E1–8, elution fractions; M, molecular weight marker. **c** Complex formation of DP1 and His-tagged DP2CTD analyzed by SDS-PAGE (left) and Native PAGE (right). Purified DP1 and DP1-His-tagged DP2-CTD complex (10 pmol) were subjected to SDS-10% PAGE, or Native-5% PAGE, followed by Coomassie Brilliant Blue staining. Protein size markers were run in lane M and their sizes are indicated on the left side of the gel

The 3D structure modeling of the C-terminal domain of DP2 (DP2CTD), which was truncated for the crystallization of *P*. *abyssi* DP2 [17], are shown in Fig. 3. After fitting the crystal structures of both DP1 and DP2 into the EM map of the PolD-PCNA-DNA complex, we still found excessive density area in the EM map. This area consisted, mainly, of a rod-shaped density and was located between DP1 and DNA (Fig. 3a and c). This extra density, most likely, can be attributed to the DP2CTD. Our yeast two-hybrid analysis revealed that DP2CTD, and in particular, the region encompassing Val1000–Lys1205, is responsible for interaction with DP1 (Fig. 4a). Furthermore, purified DP1 co-eluted with His-tagged DP2CTD from the Ni-bound fractions (Fig. 4b) and a stable complex of DP1 and DP2CTD was also detected by gel shift assay using a native PAGE (Fig. 4c). Interaction of DP1 with the C-terminal region of DP2 is consistent with the previously observed interaction in the biochemical analysis of *P*. *horikoshii* PolD [31, 32], and also, more recent cryo-EM structure of *P*. *abyssi* PolD showed the binding of DP1 to the C-terminal region of DP2. This work also showed that DP1 and the DP2CTD was co-purified and also showed the effect of deleting the DP2CTD on the catalytic activity of PolD [21].

Interaction modes of the largest catalytic subunit and the second subunit of the human Polα and Polε have been reported by the co-crystal analysis (PDB: 4Y97 for Polα and PDB: 5VBN for Polε) [33, 34]. The 3D structures of the C-terminal regions of Polα and Polε, consisting of three α-helices and two Zn-finger-like structures, are highly conserved between Polα and Polε, and there seems to be a common interaction model between the catalytic and second subunits of the eukaryotic replicases. There are limited amino acid sequence similarity between DP2CTD and the CTD of Polα p180 (22.8%) or DP2CTD and the CTD of Polε p261C (34.6%) as shown in Additional file: Figure S9. However, the secondary structure prediction suggested that the DP2CTD, which interacts with DP1, consists of four α-helices (α’, α1, α2, and α3) and a single Zn-finger-like structure (inserted between α1 and α2). This structural feature after α1 (i.e., α1, β1, β2, α2, and α3) is similar to that of the C-terminal domains (CTD) of Polα and Polε, and therefore, we built a model of the 3D structure of the DP2CTD using the CTD structures of Polα and Polε as templates (Additional file: Figure S9). The model was successfully docked into the extra bundle of rod densities together with the α’ helix of our EM density map as shown in Fig. 3b, d. In particular, the four cysteines are arranged on loops connecting the predicted helices (Fig. 3e), and density corresponding to this zinc-finger-like structure was consistently observed at the edge of this helix bundle (Fig. 3d). In contrast, the α-turn-α segment (R1006-D1034) at the C-terminus of the reported DP2 crystal (PDB: 5IJL) (Fig. 3a) is located far from the reconstructed CTD region and on the opposite side of the DNA. Moreover, the C-terminus of this segment extends in the opposite direction to DP1. Considering that most of the C-terminus-proximal region is truncated in the reported DP2 crystal and that the second α-helix of this segment protrudes from the EM map, we assume that this α-turn-α segment should be attached to the central domain (V308-G653) in a different arrangement from that in the crystal. The flexible loop between this α-turn-α segment and our constructed CTD region is 32 amino acids long which is sufficient to cover the distance (about 55 Å) between them. The cryo-EM structure of *P*. *abyssi* PolD bound to DNA showed that the reorganization of the DP2 conformation actually occurs by interaction with DP1 in *P*. *abyssi* PolD [21].

### PolD-PCNA interactions

**Fig. 5:**
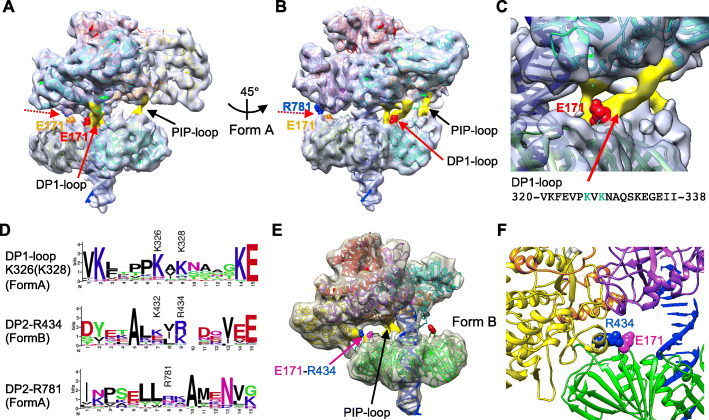
PolD-PCNA interaction. **a** Side view of Form A. The C-terminal loop of DP2, making a contact via PIP-box motif with PCNA is indicated by the black arrow. The “PIP-loop” and the “DP1-loop” are shown in yellow surface. The “switch hook” to contact with DP1 and DP2, respectively, are indicated by the red arrow and red broken arrow, respectively. The switch hook E171 is shown in red and orange sphere representation. **b** Another view of Form A, rotated by 45° around the vertical axis. The R781 of DP2 making a contact with E171 of PCNA, is shown by blue sphere representation. The E171 of two PCNA subunits used for DP1-PCNA contact are highlighted by red and orange sphere representation, **c** A closer view of DP1-PCNA contact in **a** and **b**. The loop density corresponding to K321-I338 (DP1-loop) is colored yellow. **d** Conservation of the PolD-PCNA contact sites, DP1-K326, DP2-R434, and DP2-R781. The homologous sequences of DP1 (34 *Thermococcus*, 14 *Candidatus*, 9 *Pyrococcus*, 5 *Thermococci*, and 24 other species) and DP2 (35 *Thermococcus*, 10 *Pyrococcus*, 9 *Methanolobus*, 6 *Thermococci*, and 41 other species) were aligned and applied to WebLogo [36]. **e** Side view of Form B. The C-terminal loop of DP2 making a contact via PIP-box motif with PCNA is indicated by the black arrow. The E171 used in DP1-PCNA interaction of Form A is shown in red sphere representation. A novel contact distinct from that of Form A was indicated by the magenta arrow. The third switch hook E171 is shown in magenta sphere representation. R434 of the DP2 central domain (blue sphere) is assumed to interact with the E171 residue. **f** A closer view of DP2-PCNA contact shown in **e**. The positions of the side chains are those of crystal structures and should be regarded as approximate ones

Our complex structure contained several loop densities with apparently flexible features. These loops were mainly observed to participate in the PolD-PCNA contact. Many key proteins involved in DNA replication, such as PolB, Lig, and FEN1, contain the PCNA-binding PIP-box motif sequence in their flexible terminal regions or loops. PolD also has a PIP-box motif sequence in the C-terminal loop region of DP2. A string-like density connecting PolD with PCNA was observed in a common position in the maps for Form A and Form B. This contact was located close to the C-terminus and interdomain connecting loop (IDCL), which is known as the PIP-box interacting area of PCNA for binding of the PCNA-interacting proteins [35]. This loop extended from the C-terminus of the modeled CTD of DP2 where the PIP-box motif is located. Thus, we assigned this string-like density to the PIP-loop of DP2CTD (Fig. 5 and also see Additional file: Figures S7 and S8).

In addition to this interaction via the PIP-box motif, several contacts between PolD and PCNA were observed in both forms. In the map of Form A, another contact was found in the vicinity of the PIP-box contact. Intriguingly, the amino acid residue (E171) contributing to this contact in PCNA was located in the longest turn, which protrudes from the front face of PCNA (Fig. 5a, b: red sphere). This acidic residue (Glu or Asp), referred to as the “switch hook” in our previous work [26], is highly conserved among the DNA clamps from several species and has been shown to play a key role in switching between polymerase and exonuclease modes. From the PolD side of this contact, however, a loop-like density (yellow-colored region denoted “DP1-loop” in Fig. 5c), corresponding to the disordered region between Lys207 and Ile224 in the crystal structure of *P*. *abyssi* DP1 (corresponding to Lys321-Ile338 for *T*. *kodakarensis* DP1), is hanging down to interact with the PCNA “switch hook” (Fig. 5c). Two lysine residues, K326 and K328, are located in the middle of this loop, and among these, K326 appears more frequently in the DP1-loop and, thus, is more likely to interact with E171 (Fig. 5d).

Furthermore, E171 of the adjacent PCNA subunit and R781 of DP2 (indicated by the dotted red arrows in Fig. 5a, b; E171 is indicated by orange sphere) are close enough to interact with each other. Although R781 is less conserved in evolution than other contact candidates, the adjacent amino acids also tend to be positively charged residues occurring with comparable frequencies (Fig. 5d), suggesting that these residues could serve as a potential contact point. Thus, PolD in the Form A complex appears to fix its orientation using two E171 residues of the PCNA trimer as switch hooks connected for each with DP1 and DP2, respectively.

In the Form B complex, another contact was observed between PCNA and the central domain of DP2, due to the tilt of PolD. Intriguingly, the third glutamate, E171 (Fig. 5e: magenta sphere) of the PCNA trimer, not used in Form A, was involved in this PolD-PCNA contact. A short helix of PolD was located on the PolD side of the contact, and K432 and R434 in this helix are positioned near E171. In particular, R434, which is more strongly conserved among the two (Fig. 5d), appears to be close enough to interact with E171 (Fig. 5f).

**Fig. 6:**
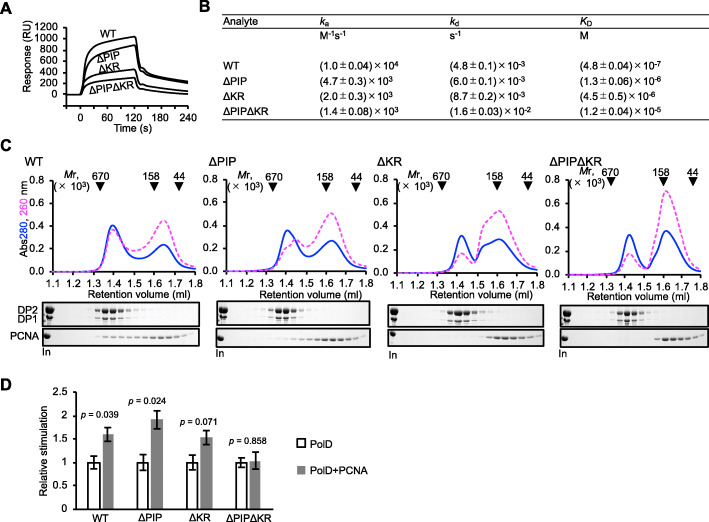
Interactions between PCNA and various PolD mutants. **a** SPR analyses were performed to detect the physical interactions of PCNA with various PolD mutants (wt, ΔPIP, ΔKR, and ΔPIPΔKR). Purified PCNA was immobilized on a sensor chip, and six different concentrations (50, 100, 200, 300, 500, 1000 nM) of purified PolDs were analyzed. The sensorgrams at 500 nM of PolDs are overlaid. **b** The kinetic parameters were calculated from the sensorgrams as shown in Figure S10. *k*a, the association rate constant; *k*d, the dissociation rate constant; *K*_D_, the apparent equilibrium constant. **c** Complex formation analyzed by gel filtration chromatography. A primed DNA composed of d29 mer (5′-GGTACCGGGCCCCCCCTCGAGGTCGACGGam-3′)/d45 mer (5′-ATCAAGCTTATCGATACCGTCGACCTCGAGGGGGGGCCCGGTACC-3′) was mixed with various PolDs and PCNA and subjected to the column. The elution profiles, monitored by the absorbance at 260 and 280 nm, are shown in the dashed red and solid blue lines, respectively. The peak positions of the marker proteins are indicated on the top. Aliquot (5 μl of injected mixture and 8 μl of each elution fraction were subjected to 10% SDS-PAGE followed by Coomassie Brilliant Blue staining. The mutant proteins are indicated by the following notations. ΔPIP, DP2(Δ1314-1324); ΔKR, DP1(K326A K328A) and DP2(K432A R434A R781A). **d** The primer extension activity of PolD variants were measured in the presence or absence of PCNA. For each PolD variant, the amount of incorporated dNTPs in the absence of PCNA was normalized to 1 and the relative activities were shown to compare the stimulation by PCNA for each PolD variant. Three independent experiments were carried out in succession for each PolD variant, and the S.E.M. values are shown as vertical lines on the plots in each graph

Notably, a similar interaction was observed between the E171 residue of PCNA and an arginine triplet on an α-helix of PolB, which was shown to play an important role in switching between the polymerase mode and the editing mode of the PolB-PCNA-DNA complex [27]. The physical contact between PCNA switch hooks (E171) and the basic residues of PolD were supported by the Surface Plasmon Resonance (SPR) analysis (Fig. 6a, b and Additional file: Figures S10) and gel filtration assays (Fig. 6c). These results suggested that the complex stability resulted not only from the interaction with the PIP-motif, but also with the additional, previously unknown contact residues (K326 and K328 in DP1, and K432, K434, and R781 in DP2) interacting with the switch hooks (E171) in PCNA. The quantitative analyses by SPR also showed that the PCNA E171-mediated interaction may be stronger than the regular interaction via PIP-box motif (Fig. 6a, b and Additional file: Figures S10). To confirm whether these interactions are actually related to function, an in vitro primer extension assay was performed using PolD and its mutants at PCNA-interaction sites. As shown in Fig. 6d, the strand synthesis activity of both single mutants of PolD, ΔPIP and ΔKR, were stimulated to the same extent as that of the wild type PolD in the presence of PCNA. However, this PCNA-dependent stimulation was not observed with the ΔPIPΔKR double mutant. This result shows that the E171-mediated interaction of PCNA affects function of PolD, as well as the interaction at PIP motif, common for PCNA-binding proteins.

### PolD-DNA interaction

**Fig. 7:**
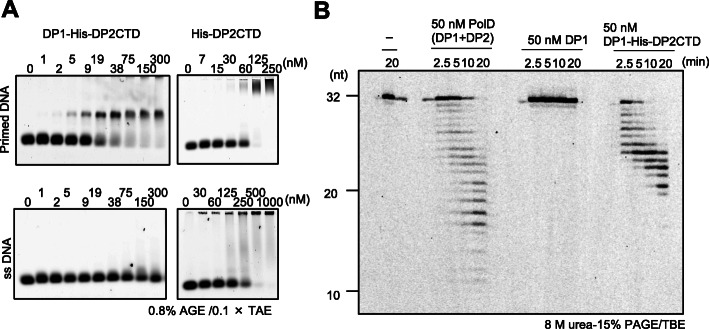
DNA binding activity and the 3′–5′ exonuclease activity of DP1-His-DP2CTD complex. **a** Electrophoretic mobility shift assay of DNA binding activity of DP1-His-DP2CTD complex and His-DP2CTD, using primed DNA (upper) and ssDNA (lower) as probes. The protein concentrations in the reactions are indicated at the top of each panel. **b** 3′–5’exonuclease activity assay. The samples and reaction time were indicated on the top of the panel. The gel images were visualized using a Typhoon Trio+ imager (GE Healthcare)

We showed that *T*. *kodakarensis* PolD and DP2, but not DP1, has binding affinity to dsDNA and primed DNA by a gel shift assay previously [20]. Here, we showed that DP2CTD by itself strongly binds DNA (Fig. 7a). The EM structure supports the direct contact of DP2CTD with DNA. The 3′–5′ exonuclease activity was not detected from DP1 alone [20], but was clearly shown in the presence of DP2CTD (Fig. 7b). Our EM structure is compatible with this explanation because DP1 does not contact DNA directly, and its proximity to DNA is based on DNA binding by DP2CTD. By contrast, it is unclear why the DNA polymerase activity was not observed with DP2 alone although DP2 strongly binds DNA [20]. DNA binding of DP2CTD is readily detectable but is weaker than that of DP1-DP2CTD (Fig. 7a). Thus, it appears likely that DP2CTD changes its conformation by forming a complex with DP1 to stabilize the complex with DNA. Indeed, there is a difference between the structure of DP2 in our 3D structure and the crystal structure of *P*. *abyssi* DP2 without the CTD [17]. In the recent structural analysis of *P*. *abyssi* PolD, large interdomain rearrangements are also observed between the DP2 crystal and the cryo-EM structure of the whole molecule of PolD [21]. The structural change of DP2CTD caused by the complex formation with DP1 is probably important for polymerase activity. Thus, in Archaea, the CTD is crucial for the functions of PolD, especially, for binding to DNA and PCNA.

In the Form A structure, the 3′-terminus of the nascent DNA chain, where elongation takes place, comes close to the polymerase active site of DP2 (Additional file: Figure S11A). Thus, we concluded that this form corresponds to the synthesis mode. The distance between the 3′-terminus of DNA and the polymerization active site (Asp965) of DP2 in our structure was about 13 Å, which is consistent with the reported 15 Å in *P*. *abyssi* PolD-DNA complex [21]. A similar movement of the N-terminal domain and rotation of the DNA around its axis, which would reduce the distance, have been postulated in the *P*. *abyssi* PolD-DNA study [21].

The comparison of DNA binding between Form A and the reported PolD-DNA complex structure [21] is shown in Additional file: Figure S11B. Although the dsDNA was bound to DP2 in a similar configuration, a rotation of 11° was observed. While the orientation of the dsDNA in the reported PolD-DNA complex from *P*. *abyssi* causes a collision between DNA and the inner wall of the PCNA channel, the dsDNA of our Form A passes through the center of the channel vertically without interacting with the inner wall (Additional file: Figures S11B and C). This conformation is suitable for the sliding motion of the PCNA clamp during DNA replication.

**Fig. 8:**
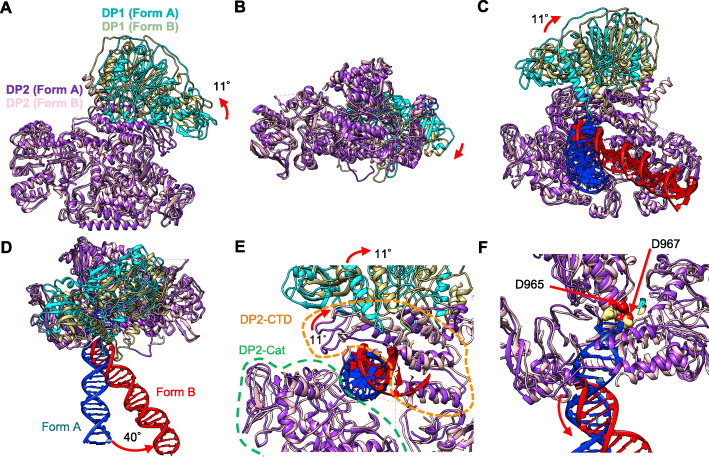
Comparison of Form A and Form B. The atomic model of PolD was aligned by superimposing the DP2 (except the modeled CTD) region of Form A (purple) and Form B (pink). **a** Top view. **b** Side view. **c** Bottom view. **d** Side view of both forms. (DP1 (Form A), cyan; DP1 (Form B), khaki; DP2 (Form A), purple; DP2 (Form B), pink; DNA (Form A), blue; DNA (Form B), red. The DNA duplex rotates 40° in the conversion from Form A to Form B. **e** Enlarged view of the DNA-PolD interacting region (bottom view). The modeled DP2-CTD (orange broken line) region rotates about 11° in conjunction with DP1. The catalytic domain of DP2 (DP2-Cat) is indicated by the green broken line. **f** Enlarged view of the DNA-PolD interacting region (side view). The active site residues D965 and D967 are colored gold (Form A) and red (Form B). The positions of the side chains of active site residues are those of crystal structures and should be regarded as approximate ones. Note that the PCNA region of the model is omitted for clarity (**a–f**)

The atomic models of DP2 are well superimposed between Form A and Form B, demonstrating the identity of the DP2 structures (Fig. 8a, b). However, the DP1-DP2 arrangements are different between the two forms. An 11° rotation of DP1 was observed, suggesting that the interaction with DNA and PCNA induced a conformational change of the PolD complex. Figure 8c and d show the comparison of the DNA backbone architectures between the two forms. The DNA in Form B was bound to DP2 in a substantially different manner from that in Form A, and the orientation of the DNA bound to DP2 differs by 40° between the two forms. This swing motion appears to pull the growing end of the DNA out of the polymerase active site (Fig. 8e, f). Moreover, the nascent chain in Form B is unwound by five bases from the 3′-terminus. These results suggest that Form B corresponds to a state distinct from the synthesis mode and thus an editing mode. Notably, in Form B, DP2CTD shifts in conjunction with the rotation of DP1, resulting in broadening of the cleft between the modeled CTD and the catalytic domain of the DP2, which clamp the DNA duplex from both sides (Fig. 8e, f).

## Discussion

Here, we present the 3D structure of PolD, the archaeal-specific DNA polymerase, complexed with a primed DNA and PCNA. PCNA is the sliding clamp that enables high processivity of DNA polymerases and thus is an essential factor for replication. This complex structure is the first step to elucidating the complete architecture of the archaeal replisome.

**Fig. 9:**
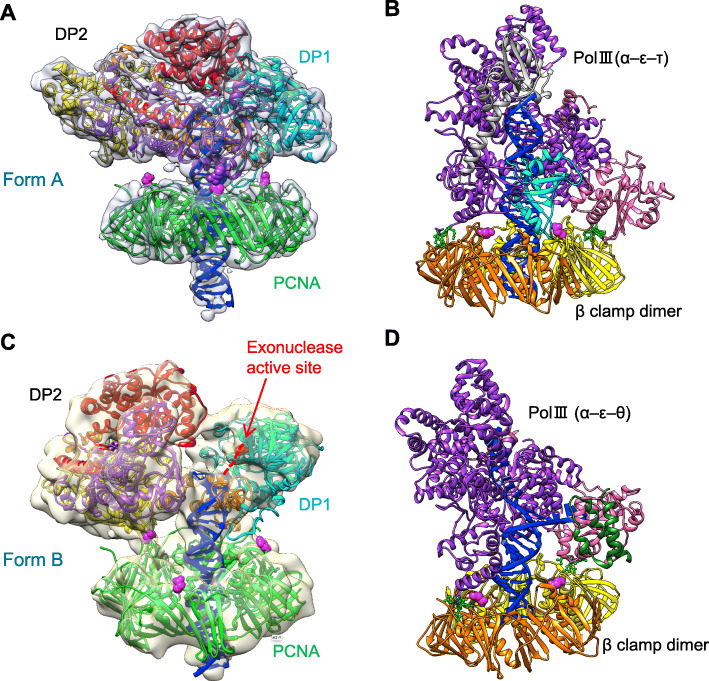
Comparison with bacterial complexes for synthesis and editing modes. **a** Form A of the archaeal complex. Each protein component is indicated and DNA is shown dark blue. E171, the proposed hook, is highlighted by magenta. **b** The atomic model of the bacterial PolIII (α–ε–τ)-β clamp complex in the synthesis mode from PDB: 5FKV [29]. The α and ε subunits are shown in purple and pink, respectively, and the OB-domain in the α subunit is highlighted by light blue. DNA was shown in dark blue. The two subunits of β clamp are shown in orange and yellow, and E276, the candidate residue of the hook on each subunit, is shown by magenta. The clamp binding motifs, corresponding to the bacterial PIP, of α and ε were shown in green balls and sticks. **c** Form B of the archaeal complex. The path to the exonuclease active site is indicated by the broken red line. **d** The atomic model of the bacterial PolIII α–ε–θ complex in the editing mode on the β clamp from PDB: 5M1S [30]. The complex undergoes a large structural conversion from the synthesis mode. The colors indicate the same components as in (**b**). The θ subunit is shown in green and E276 is highlighted by magenta

**Fig. 10:**
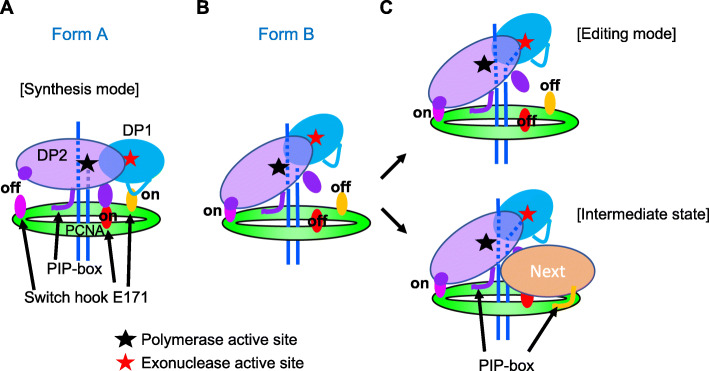
The switching hooks of PCNA for fixing conformation of PolD. **a** The Form A observed in our EM analysis. **b** The Form B observed in our EM analysis. The 3′-terminus of DNA can reach the active center of the exonuclease in DP1. The Form A and Form B can correspond to synthesis mode and editing mode, respectively. **c** The Form B can also form an intermediate state in the switching process from PolD to the next factor on the PCNA ring

The overall structure of the *T*. *kodakarensis* PolD is similar to the recently published structure of *P*. *abyssi* PolD [21]. We obtained two structures, Form A and Form B, for the PolD-PCNA-DNA complex. The two forms are structurally distinct. In Form A, the 3′-terminus of the DNA is located in the active site of DP2 to extend the DNA strand. On the other hand, the DNA orientation is toward DP1, and the 3′-terminus of the DNA can reach the active center of the exonuclease in Form B, although the 3′-terminus was not observed in the EM structure. The cryo-EM structure of *E*. *coli* PolIII (α, ε, τ)-β (sliding clamp, a PCNA homolog)-DNA (synthesis mode) [29] and PolIII (α, ε, θ)-β-DNA (editing mode) [30] have been reported, and we compared our two forms with these bacterial replicase structures (Fig. 9). Similar DNA orientations relative to the clamp molecule were observed between the archaeal and bacterial complexes in the synthesis mode (Fig. 9a, b). Focusing on the DNA in our Form B structure, the distance between the dsDNA end and the active site of the DP1 exonuclease is about 30 Å (Fig. 9c). This length is longer than that in the bacterial PolIII editing complex, in which the 3 bp strand separation at the 3′-terminus of the DNA enabled the synthesized strand to reach the active site of the exonuclease in the ε subunit (Fig. 9d). However, we could only visualize 25 bp out of the 30 bp of the dsDNA used for the present study, and five bases are predicted to be unwound from the 3′-terminus in our structure. This 5 bp unwinding would be sufficient to cover the 30 Å distance and put 3′-terminal nucleotide in contact with the exonuclease active site of DP1 (Fig. 9c). Indeed, the exonuclease activity of PolD is strong in vitro, with efficient cleavage even of mismatch-free DNA substrates [20] (see also Fig. 7a and Additional file: Figure S1) that were used in this structure analysis. Thus, it appears likely that a considerable portion of the PolD-PCNA-DNA complexes were in the editing mode in solution (Fig. 9c). Therefore, we infer that the two forms we obtained represent the synthesis and editing modes of the archaeal replisome, respectively (Fig. 10a, c).

From these two forms, we found the novel interactions between PolD and PCNA in addition to the conventional PIP motif-mediated interaction. The E171 is the key residue in PCNA for the newly identified interactions. We propose here the mechanism for a locking conformation of PolD on PCNA ring using E171s as hooks. Three E171 hooks on the PCNA trimer ring can work for interactions. In Form A, two hooks, one for each DP1 and DP2, are on and another hook for DP2 is off (Fig. 10a). On the other hand, the on/off state of these three hooks is reversed in Form B (Fig. 10b). The reasonable interpretation of these structures is that PolD conformation is fixed in the synthesis mode on the PCNA by fastening the two E171 hooks with both DP1 and DP2, and the conformation can be converted to the editing mode by releasing the above two hooks and hanging the third hook at different site on DP2. It can be predicted that synthesis mode with two hang-on hooks is more stable than editing mode with one hang-on hook. Actually, we obtained the Form A particles much more than the Form B particles in our EM images. The stabilization of the synthesis mode is advantageous for the replicase to perform the processive strand synthesis. We confirmed that the interaction of PolD at E171 of PCNA is critical for the complex stability and DNA synthesis activity in vitro by mutational analysis (Fig. 6). However, further studies are needed to elucidate the detailed mechanism on how to switch the synthesis and editing modes of PolD on PCNA.

The hook on the clamp molecule might be conserved in the replisome of the three domains of life. We examined the reported structure of the bacterial replicase [29] and found that the Glu276 residue at the center of a loop in the β subunit can interact with the OB-domain of the α subunit in the synthesis mode of PolIII (Fig. 9b), while the OB domain was invisible in the reported editing complex [30], as shown (Fig. 9d), implying the detachment of this domain from Glu276 on β clamp. These observations support that Glu276 may function as the hook in the bacterial clamp. However, that substantial protein interfaces are present between the bacterial PolIII and β-clamp, as compared with the archaeal PolD-PCNA in both Forms A and B, in which only two or three sites are involved. This finding supports that the replication machinery of Bacteria and Archaea/Eukarya are derived from different origins, and the role of the glutamate hook may be diversified. The Glu residue corresponding to E171 of the archaeal PCNA is highly conserved in eukaryotic PCNAs (E174 in human PCNA, PDB: 1AXC) [37], suggesting conservation of the switch hook function. Our previous studies on *P*. *furiosus* PolB-PCNA-DNA [26, 27] showed that E171 of PCNA seems to work as a hook to lock the conformation of PolB, the eukaryotic-like family B DNA polymerase, in synthesis mode or editing mode on PCNA.

PolD covers the front face of PCNA and occupies the binding surfaces in the Form A complex. Thus, Form A appears to block the access of other PCNA-binding proteins. In contrast, PolD is substantially tilted on the PCNA in the Form B complex, and one of the three PCNA subunits is available to accept other factors. The dsDNA also appears to be slightly shifted to the edge of PolD. This conformation seems to suggest that other factors might approach DNA from the opposite side of PolD in Form B (Fig. 10c). To investigate whether PolD could coexist with other replication factors on the same PCNA ring, the crystal structure of human FEN1-PCNA (PDB: 1UL1) was overlaid on both maps (Additional file: Figure S12). In this crystal structure, three FEN1 molecules were bound to three different subunits of the PCNA ring in distinct orientations (denoted X, Y, Z, respectively) [38]. FEN1 in the most flipped-out X orientation can coexist with PolD in the Form A structure (Additional file: Figure S12A). By contrast, FEN1 can coexist without colliding with PolD on the PCNA in the Form B in any orientation of X, Y, Z (Additional file: Figure S12B-D). In particular, our recent study of the FEN1-PCNA-DNA complex showed that the Y orientation of FEN1 is closest to the functional structure [28]. Furthermore, the structure of the FEN1-Lig-PCNA-DNA complex also revealed that this complex encloses DNA from both sides with FEN1 and DNA ligase, and we proposed it as the intermediate state where DNA is being handed over from FEN1 to DNA ligase [28]. Our present structure showed similar arrangements of PolD, PCNA, and DNA, and docking examination supported our scenario in which DNA is transferred from DP2 (polymerase) to DP1 (editing exonuclease).

This structural arrangement of PolD on PCNA appears conducive to the transfer of the DNA from strand synthesis to another event in replication, and other possibilities are still open for the functional mode of Form B complex. For example, the current understanding is that PolD functions in both leading and lagging synthesis during archaeal DNA replication and that DNA should be transferred from PolD to FEN1 for Okazaki fragment processing in lagging synthesis. It would be expected that FEN1 is recruited onto the PCNA after finishing DNA synthesis and conversion of PolD on PCNA from Form A to Form B. In this case, Form B might be the strand displacement (SD) mode converted from Form A by elongation stall because of reaching the Okazaki fragment in front. The SD activity of PolD separates the front strand containing the RNA primer from the template strand, creating a flap structure, which can be cleaved by FEN1 for Okazaki fragment maturation. An in vitro study presented that PolD from *Thermococcus* species 9° N halted its DNA strand synthesis before strand displacing the downstream Okazaki fragment, and the authors proposed that PolB comes to work to strand displacement of the 5′-region of the downstream fragment to form a flap structure [39]. In this case, PolB may be recruited to the space on PCNA in Form B. In addition, if nascent DNA synthesis is stalled by a lesion in template DNA, PolD could switch to Form B, and a translesion polymerase might join the complex through interaction with PCNA (although translesion synthesis has not yet been identified in *T*. *kodakarensis*). Other repair proteins could be recruited to function in alternative repair pathways for restarting DNA synthesis. Therefore, exposure of one of the PCNA subunits in Form B appears advantageous for recruitment of additional PCNA-binding proteins to perform functions different from DNA synthesis.

PolD is an enzyme unique to Archaea, and no homolog is found in Bacteria or Eukarya. The evolutionary relationships between the archaeal and eukaryotic replicases appear to be highly complex. DP1 is homologous to the second subunits of the eukaryotic Polα, Polδ, and Polε that, however, have lost the exonuclease activity [17], which in Eukarya was relegated to the nuclease domains of the respective large subunits. By contrast, DP2 shows no significant sequence similarity to other proteins although the recent structural analyses of *P*. *abyssi* PolD revealed the structural similarity between DP2 and the DPBB RNA polymerase family suggesting an ancient common origin of the polymerases involved in transcription in all three domains of life and in replication in Archaea [17, 21]. It is of further note that the DP2CTD shares a conserved Zn-finger-like motif [9] and shows structural similarity with the CTDs of the catalytic (largest) subunits of Polα and Polε [21]. Our independent analysis of *T*. *kodakarensis* PolD also showed that the 3D structure model of DP2CTD, built by using the CTDs of the catalytic subunits of Polα and Polε as templates, fitted well to the density map of our EM structure. Thus, at the origin of eukaryotes, the archaeal PolD DP2 was apparently replaced by PolB but the CTD was retained.

During manuscript review, a report of a similar structure analysis was published, in which the Sauguet group showed the cryo-EM structure of the DNA-bound PolD-PCNA complex from *P*. *abyssi* at 3.77 Å resolution [40]. Their structure is similar to our Form A. The authors identified two different PIP boxes in PolD to interact with PCNA. In addition to canonical PIP (cPIP), which is an actual PCNA-binding site in DP2 of *P*. *furiosus* and *P*. *abyssi* [22, 41], the authors found a second PIP located at an internal site from cPIP, and they called it internal PIP (iPIP). The authors found that iPIP, not cPIP, is important for the functional PolD–PCNA interaction, especially in the presence of DNA. In addition, the authors proposed that cPIP plays a dual role in binding either PCNA or primase and could be a master switch between an initiation and a processive phase during replication. The amino acid sequence of the C-terminal region of DP2 is also conserved in *T*. *kodakarensis* DP2. Unfortunately, as the two PIPs, 40 amino acids apart from each other, are located on the same flexible loop, it was difficult to discriminate which of the two binds to PCNA in our 6.9 Å structure. The structure data of *P*. *abyssi* complex in PDB was released recently, and we did re-modeling of the C-terminal region (E1204-S1228) of DP2 based on the *P*. *abyssi* DP2 [40], which was made by the EM map with a resolution higher than ours. Further analyses are necessary to understand structure and functions of the C-terminal region of DP2 in more detail. In this study, we showed that the PIP region is involved in PCNA binding, and in addition, a new PCNA-binding site in *T*. *kodakarensis* DP2, discovered in this study, is different from either cPIP or iPIP. Another PCNA-binding site at the N-terminal region of *P*. *abyssi* DP2, reported previously [41], is also different from the sites we found in *T*. *kodakarensis* DP2 in this study. Further studies will make these different PCNA binding sites clearer for relation to the functions.

A report showing the cryo-EM structures of human Pol δ-PCNA-DNA-dTTP complex at 4.1 Å has also been appeared during our manuscript review [42]. The authors showed three different conformers of the complex, in which the orientation of the PCNA ring to Polδ varies in a range up to 20°. The catalytic subunit, p125, contacted with one of the three PCNA protomers at three sites including a PIP-box motif in the C-terminal region in the most popular conformation. However, only the interaction via the PIP-box was maintained in the other conformations, notably the interactions important for the processive synthesis was disrupted. It is especially interesting that the authors also obtained the complex structure including FEN1 on the same PCNA ring, suggesting the efficient handoff of DNA from Polδ to FEN1 for the Okazaki fragment maturation. There is not detailed description of the editing mode in this report. Both active centers for synthesis and editing of Polδ are located in the same polypeptide, p125. If the open conformation of Polδ on PCNA to allow simultaneous binding of FEN1 observed in this report is actually the synthesis mode, the mechanism for switching from synthesis to editing in the eukaryotic replicase may be different from that in Archaea. Further structural analysis would be interesting.

## Conclusions

PolD is the archaea-specific DNA polymerase that functions as the replicase in most of the organisms in Archaea except for Crenarchaeota. We isolated the PolD-PCNA-DNA complex from the hyperthermophilic archaeon *Thermococcus kodakarensis* and determined its 3D structure using single-particle cryo-EM. Analysis of the archaeal replisome structure reveals secondary interaction sites between PolD and PCNA, in addition to the canonical interaction via the PCNA-interacting protein box. Furthermore, we identify a glutamate residue that may be involved in these secondary interactions and seems to function as hooks to lock the PolD conformations for DNA synthesis and editing modes, respectively, on PCNA. Further investigation will elucidate if the proposed hook will actually regulate the functional modes of the replicase on PCNA. The conformational change of the replicase on PCNA probably contributes to the functional regulation of the replisome, which is dynamically moving during DNA replication process.

## Methods

### Protein expression and co-purification

The wild-type DP1, DP2, and PolD from *T*. *kodakarensis* were prepared as described earlier [20]. DP1 and His-tagged DP2CTD (1000-1324) were produced in combination with *Escherichia coli* BL21-CodonPlus (DE3)-RIL cells (Agilent Technologies, Santa Clara, CA). *E*. *coli* cells were cultured at 37 °C in Luria-Bertani medium, containing 50 μg/ml ampicillin, 34 μg/ml chloramphenicol, and 50 μg/ml kanamycin, to an OD_600_ = 0.3. Gene expression was induced by adding IPTG to a final concentration of 1 mM and cells were cultured at 25 °C for 16 h. The cells were collected, resuspended in buffer A, containing 20 mM HEPES, pH 7.5, 1 M NaCl, and 10 mM imidazole, and were disrupted by sonication, followed by heat treatment at 80 °C for 20 min. The soluble fraction was applied to the affinity column filled with TALON resin (Takara Bio, Shiga, Japan), and the column was washed with buffer A. The bound fraction was eluted with 20 mM HEPES, pH 7.5, 1 M NaCl, and 150 mM imidazole. Further purification procedure was described earlier with some modifications [20]. PolDΔPIP (DP2Δ1314-1324), PolDΔKR (DP1 K326A/R328A, and DP2 K432A/R434A/R781A), and PolDΔPIPΔKR were prepared by the recombinant *E*. *coli*, containing the corresponding genes, in which each mutation was introduced by PCR-mediated site-directed mutagenesis. Each designed mutation was confirmed by nucleotide sequencing. The primers used for each mutagenesis are as follows: TK1903_R781A_F; 5′-GCCCGCCGACCTGTTAGCGCAGGCCATGGACAACC-3′, TK1903_ R781A_R; 5′-GGTTGTCCATGGCCTGCGCTAACAGGTCGGCGGGC-3′, TK1903_K432R434A_F; 5′-GACTATGAAACGGCTCTAGCGGTCGCGAACGAGGTTGACGAGATC-3′, TK1903_K432R434A_R; 5′-GATCTCGTCAACCTCGTTCGCGACCGCTAGAGCCGTTTCATAGTC-3′, TK1902_K326R328A_F; 5′-GAAGTTCGAGGTTCCCGCGGTCGCGAACGCCCAGAGCAAGG-3′, TK1902_K326R328A_R; 5′-CCTTGCTCTGGGCGTTCGCGACCGCGGGAACCTCGAACTTC-3′.

For the purifications of these PolDs, TOYOPEAL Phenyl 650S (TOSOH, Tokyo, Japan), HiTrap Heparin HP (GE Healthcare, Little Chalfont, UK), and BioPro IEX SmartSep Q10 (YMC) were used for the column chromatography. *T*. *kodakarensis* has two PCNAs, PCNA1 and PCNA2, and PCNA1 is the essential clamp molecule in the cells [23]. Therefore, PCNA1 is called TkoPCNA in this study. The purification of TkoPCNA was done as described previously [23].

### Preparation of PolD-PCNA-DNA complex

We reconstituted the PolD-PCNA-DNA complex by mixing the purified TkoPolD and TkoPCNA proteins, with two kinds of primed DNA, which were prepared by annealing two deoxyoligonucleotides shown in Additional file: Figure S1 (synthesized by Hokkaido System Science, Hokkaido, Japan and Sigma-Aldrich Japan, Tokyo, Japan) in 20 mM Bis–Tris, pH 7.0, and 50 mM NaCl, and incubated at 37 °C for 15 min. To prevent degradation of the primed DNAs by the 3′–5′ exonuclease of PolD, four successive phosphorothioate modifications were introduced into the 3′-terminus of both strands. The purified TkoPolD, TkoPCNA, and primed DNA (30/45) (the sequences are shown in Additional file: Figure S1) were mixed and incubated in a solution containing 50 mM Tris–HCl (pH 8.0), 300 mM NaCl, 1 mM Dithiothreitol, 0.1 mM EDTA (without MgCl_2_) at 20 °C for 15 min. The reconstituted PolD-PCNA-DNA (30/45) complex was loaded onto a Superdex 200 5/150 (GE Healthcare) gel filtration column equilibrated with a solution of the same composition, containing NaCl at 50 mM instead of 300 mM. For the preparation of the PolD-PCNA-DNA (25/35) complex, the mixture was loaded onto the same column, but the equilibrium and elution buffer contained 150 mM NaCl, instead of 50 mM. The protein compositions of both complexes, eluted at the peak fractions, were analyzed by SDS-PAGE.

### Electron microscopy and single-particle image analysis

For the negatively stained specimen, a 3-μl aliquot of sample solution was applied to a glow discharged, continuous thin-carbon film supported by a copper grid, left for 1 min, and then stained with three drops of on-ice-cooled 2% uranyl acetate. Stained samples are examined by a Tecnai T20 electron microscope (FEI, Hillsboro, OR) operated at 200 kV accelerating voltage. Images were recorded using an Eagle 2k CCD camera (FEI). Holey carbon (Quantifoil R1.2/1.3 Au 200) grids were used for frozen-hydrated specimens. Grids were glow discharged for 1 min by an HDT-400 hydrophilic treatment device (JEOL, Tokyo, Japan) before usage. Two-three μL of sample solutions were applied on holey grids for rapid freezing. Rapid freezing was performed using EM-GP (Leica Wetzlar, Germany) or Vitrobot (FEI) freezing robots.

### Cryo-EM data collection

All the frozen-hydrated samples were first examined by a Polara electron microscope (FEI) operated at 200 kV accelerating voltage, to optimize the sample preparation conditions. Images were taken using either an UltraScan 2 k CCD camera with a Gatan GIF Tridiem energy filter (GATAN, Pleasaston, CA) or an UltraScan 4 k CCD camera (GATAN). Electron microscopic image datasets of PolD-PCNA-DNA (30/45) complex for structure analyses were collected on a Titan Krios electron microscope (Thermo Fisher Scientific, Waltham, MA), equipped with a spherical aberration (Cs) corrector (CEOS GmbH, Heidelberg, Germany), under 300 kV accelerating voltage. An EPU software (Thermo Fisher Scientific) was used for fully automated data collection. Images were recorded by Falcon II direct electron detector (Thermo Fisher Scientific), in the dose-fractionation mode (2 s exposure/32 fractions), at a nominal magnification of × 59,000, corresponding to 1.10 Å/pixel on the specimen. The nominal defocus was in the range of − 1.0 μm ~ − 1.4 μm. A total electron dose of 36 electrons/Å^2^ was used for each image recording. A Volta phase plate (VPP) was used to enhance the contrast of particle images. Electron microscopic images of the PolD-PCNA-DNA (25/35) complex for structure analysis were collected on a Talos Arctica electron microscope (Thermo Fisher Scientific) using a Falcon 3EC direct electron detector (Thermo Fisher Scientific). Images were recorded in the electron counting mode, at a nominal magnification of × 92,000, corresponding to 1.12 Å/pixel on the specimen, with 47 s total exposure time. The nominal defocus range of the data was − 1.0 μm ~ − 3.0 μm. Intermediate frames (total 1821 movies) were recorded every 0.64 s, giving an accumulated dose of 40 electrons/Å^2^ and a total of 74 fractions per image (i.e., 0.54 electrons/Å^2^ dose per fraction).

### Cryo-EM image processing

Movie frames of PolD-PCNA-DNA (25/35) and PolD-PCNA-DNA (30/45) were aligned to correct the dose-induced and dose-weighted motions of the specimens using MotionCor2 [43]. The contrast transfer function was determined for each image using the CTFFIND4 program [44]. About one thousand particle images were picked manually from the images using the Relion [45] manual picking tools. This initial small dataset was subjected to a reference-free 2D classification in Relion. Selected “good” class average images were used as references to automatically pick particles, using Gautomatch (http://www.mrc-lmb.cam.ac.uk/kzhang/Gautomatch/). The output coordinates of the picked particles were used for the particle extraction program of Relion [45].

A total of 240,256 particles were extracted from the PolD-PCNA-DNA (30/45) complex images, and after 2D classification procedure, particles classified as “bad” were removed from the data set. In total, 171,739 particles were subjected to the 3D classification procedure in Relion (Class3D), assuming eight classes. Particles classified as the best (class 8, 30,889 particles) and second-best (class 1, 19,356 particles) 3D classes were further used for refining (Refine3D in RELION) each of the two class of 3D maps. The refined map of class 1 (corresponding to the Form B), was sharpened by applying a negative B-factor (− 150) and corrected for the MTF of the Falcon II detector.

The extracted 309,113 images of the PolD-PCNA-DNA (25/35) complex were subjected to 2 turns of reference-free 2D classification (Class 2D, Relion) and particle selection procedure to remove bad images. In total, 230,965 images of the PolD-PCNA-DNA (25/35) complex were subjected to 3D classification procedure in Relion assuming eight classes of 3D structures. The initial volume for this procedure was obtained by the “3D initial model” procedure in Relion. Among the eight classes, only one 3D class (Class 2) corresponding to the Form A, exhibited interpretable high-resolution structure. Also, 41,073 particle images, assigned to this class, were used for “3D auto refinement” in Relion. After refinement, “Movie refinement” and “Particle polishing” were applied to improve resolution. The final map was obtained after 2nd “3D auto refinement” and “Post Process” which includes the 3D masking, correction for the MTF of the Falcon 3EC detector, and B-factor sharpening (*B* = − 500). The resolution of the final maps was estimated by Gold standard FSC using FSC = 0.143 criteria.

### TkoPolD-PCNA-DNA model construction

The model coordinates of the TkoPolD-PCNA-DNA complex were constructed by assembling several crystal structures and applying the homology modeling method. The crystal structures of *P*. *abyssi* DP1 (PDB: 5IHE) and DP2 (PDB: 5IJL) [17] were first fit into the density map of Form A as described in the text. Then, the crystal structure of the TkoPCNA trimer (PDB: 3LX1) and a model of primed DNA (30/45) in standard B-form parameters were fit into the density map [46].

Next, the parts of the model, which were not presented in the above-mentioned close homologs, were constructed. A density predicted to be a helix bundle was observed at DP1-DP2 interface, which was presumed to be similar to that of human Polα B-subunit–Polα catalytic subunit (PDB: 4Y97) or human Polε subunit 2–catalytic subunit A (PDB: 5VBN) interfaces [33, 34]. Thus, Phe1322-Phe1440 of Polα catalytic subunit (chain B of 4Y97) was introduced into the model by superposing DNA polymerase B α-subunit (chain A of 4Y97) on the DP1 subunit. A density predicted to be a short helix was observed at the PIP-binding site of PCNA. TkoDP2 had PIP-box peptide (1316-ISLDEFFGS-1324) at the C-terminus, which was similar to that of human RNase H2 subunit B (295-KSIDTFF-301; chain B of PDB: 3P87) or DNA annealing helicase and endonuclease ZRANB3 (518-KQHDIRSFFV-527; chain B of PDB: 5MLO), in which the consecutive Phe residues provided most of the interactions with the PCNA [47, 48]. Thus, the PIP-box peptide (chain B of 3P87) was introduced into the model by superposing PCNAs (chain A of 3P87 upon chain E of the *T*. *kodakarensis* model).

Then, the sequences of the modeled parts were converted into that of *T*. *kodakarensis* subunits via homology modeling using MODELLER [49]. The model coordinates were further refined by using PHENIX suite [50], repeatedly applying real-space refinement by using phenix.real_space_refine [51], manual model refinement by using COOT [52], and geometry optimization by using phenix.geometry_minimization. The residues absent from the original model and mainly forming helices were added to the model where they were consecutive to the modeled parts if significant densities were observed. They were consistent with secondary structure predictions, namely, Asn319–Val327 of DP1, and Ile331–Pro346, Val454–Asn457, Arg1007–Val1018, Gly1080–His1103, and Thr1282–Arg1294 of DP2. The model finally showed a map correlation coefficient of 0.76 for whole unit cell (0.70 for masked region), and the MolProbity score of 1.87, with 0% Ramachandran outlier, 0.15% rotamer outlier, and 16.03 clash score [53]. The unmodeled segments in the final model were the N-terminal domain (Met1–Ala285) of DP1, and Lys289–Asn314, Arg365–Thr371, Asp383–Lys399, Ala663–Met676, Arg1048–Leu1079, Glu1199–Leu1281, and Gly1295–Lys1314 of DP2. The model of Form B was constructed based on the Form A model by applying real-space refinement, manual refinement, and geometry optimization as for the Form A model. The final model showed a map correlation coefficient of 0.87 for whole unit cell (0.78 for masked region), and the MolProbity score of 1.63, with 0% Ramachandran outlier, 0.35% rotamer outlier, and 11.73 clash score (Additional file: Table S1). The coordinates of Form A and Form B models have been deposited to the Protein Data Bank with the accession codes 6KNB and 6KNC, respectively.

### Yeast two-hybrid assay

A yeast two-hybrid (Y2H) detection system (Matchmaker™ Gold Yeast Two-hybrid System, Matchmaker GAL4 Two-Hybrid System 3, Takara Bio) was used to screen for DP1-DP2 interacting region. The plasmid pGBKT7, encoding the GAL4 DNA binding region, and the plasmid pGADT7, encoding the activation domains, were, respectively, used to prepare plasmids containing the gene encoding DP1 and various DP2 fragment. Co-transformations of the yeast Y2H Gold cells with pGBKT7-DP1 and the pGADT7-DP2 fragments were performed according to the manufacturer’s protocol (Clontech Matchmaker manual). Cell suspensions (3 μl of 2 × 10^6^ cell/ml) of each strain were spotted onto synthetic defined (SD) plates without Leu and Trp for non-selection plate and Leu, Trp, and His, or Leu, Trip, His, and Ade for two different selection strengths plates. The agar plates were incubated at 30 °C for 4 days, and growing cells indicated the interactions of the two proteins produced from the two plasmids used for the co-transformation.

### Primer extension assay

The primer extension ability of TkoPolD was measured by counting incorporated radioactivity into DNA strands using dNTP containing [methyl-^3^H]-dTTP as substrates, and the activities were compared among the WT, ΔPIP, ΔKR, and ΔPIPΔKR in the presence and absence of PCNA. The reaction was performed in 25 μl containing 20 mM Tris–HCl, pH 8.0, 100 mM NaCl, 10 mM KCl, 10 mM (NH_4_)_2_SO_4_, 2 mM MgCl_2_, 0.1% Triton X-100 and 0.1 mg/mL BSA, 10 nM template primer substrate (prepared by annealing M13mp18ssDNA and a deoxyoligonucleotide, M13-63; 5′-dTGCCAAGCTTGCATGCCTGCAGGTCGACTCTAGAGGATCCCCGGGTACCGAGCTCGAATTCGT-3′), 0.2 mM dNTPs including 0.13 μM [methyl-^3^H]-dTTP (PerkinElmer, MA), 20 nM PCNA, and 5 nM PolD, at 72 °C for 1, 2, and 4 min. The reaction mixture was pre-incubated for 3 min, and PolD was added to initiate the reaction. After incubation, aliquots (8 μl) were fractionated and spotted onto DE81 filters (GE Healthcare). The filters were washed with 5% Na_2_HPO_4_ solution thrice and dried. Incorporated radioactivity was measured with a scintillation counter Tri-Carb 3110TR (PerkinElmer).

### 3′–5′ exonuclease activity assay

The exonuclease reaction was performed in a 20 μl containing 20 mM Tris–HCl, pH 8.0, 10 mM KCl, 10 mM (NH_4_)_2_SO_4_, 1 mM DTT, 0.1 mg/ml BSA, 2 mM MgCl_2_, and 10 nM Cy5-labeled DNA substrates (prepared by annealing two deoxyoligonucleotides, 5′ Cy5 pri32 and temp45, shown in Additional file: Figure S1, and 50 nM of the recombinant proteins at 65 °C for indicated times). The reaction mixture was pre-incubated for 2 min, and the proteins were added to initiate the reaction. After incubation, an aliquot (4 μl) was fractionated and an equal volume of stop solution (98% formamide and 0.01% orange G) was added followed by 8 M urea-15% PAGE. The gel images were visualized using a Typhoon Trio + imager (GE Healthcare).

### Electrophoretic mobility shift assay

Electrophoretic mobility shift assay was performed in 20 μl containing 20 mM Tris–HCl, pH 8.0, 10 mM KCl, 10 mM (NH_4_)_2_SO_4_, 1 mM DTT, 0.1 mg/ml BSA, 2 mM MgCl_2_, 2, 5 nM Cy5-labeled DNA (5′ Cy5 pri32 for ssDNA, 5′ Cy5 pri32, and temp45 for primed DNA, shown in Additional file: Figure S1) substrates, and indicated concentrations of proteins at 40 °C for 10 min. The protein–DNA complexes were fractionated by 0.8% agarose gel electrophoresis in 0.1 × TAE buffer and visualized with a Typhoon Trio + imager. The binding affinities were calculated as described previously [54].

### Surface plasmon resonance (SPR) analysis

The Biacore J system (GE Healthcare) was used to test the physical interactions of PCNA with various PolD mutants. PCNA was fixed on a CM5 Sensor Chip (GE Healthcare), according to the manufacturer’s recommendations. To measure the kinetic parameters, purified PolDs (PolDwt, PolDΔPIP, PolDΔKR, and PolDΔPIPΔKR) in a running buffer (10 mM HEPES–NaOH pH 7.4, 0.15 M NaCl, 0.1% Tween 20), with six different concentrations (50, 100, 200, 300, 500, 1000 nM), were applied for 120 s to the PCNA-immobilized chip, at a continuous flow rate of 30 μl/min at 25 °C. The bound analytes were removed by washing with regeneration buffer (10 mM HEPES–NaOH pH 7.4, 1 M NaCl, 0.1% Tween 20) at the end of each cycle. The apparent equilibrium constants (*K*_D_) of the interactions were determined from the association and dissociation curves of the sensorgrams, using the BIAevaluation (ver. 4.1) software (GE Healthcare).

### Analytical gel filtration chromatography

Analytical gel filtration chromatography was performed using the SMART system (Amersham Pharmacia, Buckinghamshire, UK). PolD (wt, ΔPIP, ΔKR, ΔPIPΔKR), PCNA, and DNA (each 5.4 μM, as a heterodimer: PolD, homotrimer: PCNA in 30 μl) were mixed and incubated for 3 min at 60 °C. The protein solutions were applied to a Superose 6 PC 3.2/30 column (GE Healthcare) and were eluted with buffer containing 50 mM Tris–HCl, pH 8.0, and 0.3 M NaCl. Aliquots (5 μl) of applied solution and aliquots (8 μl) of each fraction from the eluates were subjected to 10% SDS-PAGE, followed by Coomassie Brilliant Blue staining. The standard marker proteins, including thyroglobulin (670,000), γ-globulin (158,000), ovalbumin (44,000), and myoglobin (17,000), were also subjected to gel filtration as controls.

## Supplementary information

Additional file 1: **Table S1.** Model refinement statistics. **Figure S1.** The synthetic deoxyoligonucleotides used in this study. Bases with lower case letters are linked by phosphorothioate bond for protection from the 3′–5′ exonuclease activity. **Figure S2.** Sample preparations. (A) Gel filtration chromatography of the reconstituted PolD-PCNA-DNA (30/45) complex. The absorbances at 260 nm and 280 nm are indicated by red and blue lines, respectively. (B) The peak fractions in (A) were analyzed by a gradient (10–20%) SDS-PAGE. The lanes for the purified proteins used for complex reconstitution and the markers are indicated by the notations “PCNA,” “PolD,” and “Marker,” respectively. (C) Gel filtration chromatography of the reconstituted PolD-PCNA-DNA (25/35) complex. (D) The peak fraction indicated by the asterisk (*) in (C) was analyzed by a gradient (10–20%) SDS-PAGE. **Figure S3.** Electron microscopic images of PolD-PCNA-DNA complex. (A) Representative electron microscopic image of negatively stained PolD-PCNA-DNA (30/45) complex, recorded by a CCD camera. (B) Representative cryo-electron microscopic image of PolD-PCNA-DNA complex, recorded by a CCD camera, without Volta Phase Plate (VPP). The white and magenta broken circles indicate tetrameric and pentameric clusters of the complex, respectively. (C) Representative cryo-electron microscopic image of PolD-PCNA-DNA complex, recorded by a Falcon II direct electron detector, using VPP. The white and magenta broken circles indicate tetrameric and pentameric clusters of the complex, respectively. (D) Representative cryo-electron microscopic image of PolD-PCNA-DNA complex, recorded by a Falcon 3EC direct electron detector without phase plate. The scale bars indicate 50 nm. **Figure S4.** Flow charts of 3D classification and refinement procedures for the PolD-PCNA-DNA (30/45) complex. The details are described in the Methods section. **Figure S5.** Flow charts of 3D classification and refinement procedures for the PolD-PCNA-DNA (25/35) complex. The details are described in the Methods section. **Figure S6.** EM analysis. (A) Comparison between representative 2D class averages (avg) and projections from the 3D model (prj) of the Form A complex. (B) Comparison between representative 2D class averages (avg) and projections from the 3D model (prj) of the Form B complex. Gold standard Fourier shell correction (FSC) curves for the EM density maps of the Form A complex (C), and Form B complex (D). Resolutions are given for the FSC 0.143 criteria. Euler angle distributions of particles used for the final maps of, (E) Form A complex, and (F) Form B complex. **Figure S7.** 3D map and atomic model fitting of PolD-PCNA-DNA complex (Form A). (A) Top view. The arrows with the angles indicate rotations to see the bottom view (B) and the side views of (C), (D), and (E), respectively. The PCNA-interacting C-terminal loop of DP2, harboring the PIP-box, is indicated by the red arrow in (D). The crystal structures of *T*. *kodakarensis* PCNA and *P*. *abyssi* DP1 are shown in green and cyan ribbon. The N-terminal, catalytic, center, and C-terminal domains of *P*. *abyssi* DP2 are shown in red, purple, yellow, and orange ribbon, respectively. (G) Top view of the Form A map, with the crystal structures of DP1 ((PDB: 5IHE, cyan) and DP2 (PDB: 5IJL, blue) docked as rigid bodies. (H) Top view of the Form A map in which each domain of DP2 (N-terminal (red), catalytic (purple), center (yellow), and C-terminal (orange)) is docked independently into the map. Note that the PCNA region of the map is omitted for clarity. **Figure S8.** 3D map and atomic model fitting of PolD-PCNA-DNA complex (Form B). (A) Top view. The arrows with the notation of C, D, E indicates the view angle of side views of (C), (D), and (E), respectively. (B) Bottom view. (C) – (E) Side views. The PCNA-interacting C-terminal loop of DP2, harboring the PIP-box, is indicated by the red arrow. The crystal structures of PCNA and DP1 are shown in green and cyan ribbons, respectively. The N-terminal, catalytic, center, and C-terminal domains of DP2 are shown in red, purple, yellow, and orange ribbon, respectively. (F) Oblique view of the Form B map, in which PolD is aligned in the same direction as Supplementary Figure S7G. The crystal structures of DP1 (PDB: 5IHE, cyan) and DP2 (PDB: 5IJL, blue) are docked as rigid bodies into head and body region, respectively. (G) The same view as shown in (F), in which each domain of DP2 (N-terminal (red), catalytic (purple), center (yellow), and C-terminal (orange)) is docked independently into the map. Note that the PCNA region of the map is omitted for clarity. **Figure S9.** Structural similarity between eukaryotic family B and archaeal family D DNA polymerases. (A) Structure-based sequence alignment of the C-terminal domains of p261 of Polε (p261C), p180 of DP2 (p180C) and DP2 of PolD (DP2C) were performed with reference to a previous report [55]. Amino-acid sequences, belonging to α1–3 residues, are aligned by PROMALS3D (PROfile Multiple Alignment with predicted Local Structures and 3D constraints), and colored as in Fig. 3. In the bottom line, “*” and “:” indicate identical and similar amino acid residues, respectively. (B) Crystal structure of human Polε (left, 5VBN), and model structure of *T*. *kodakarensis* PolD (right). Secondary structures of the C-terminal domains are shown in schematic representations. Zn1, Zn2, Zinc-finger; α1–3, alpha helix; β, beta sheet. **Figure S10.** Interactions between PCNA and various PolD mutants. SPR analyses were performed to detect the physical interactions of PCNA with various PolD mutants (wt, ΔPIP, ΔKR, and ΔPIPΔKR). Purified PCNA was immobilized on a sensor chip, and six different concentrations (50, 100, 200, 300, 500, 1000 nM) of purified PolDs were analyzed. The apparent equilibrium constants (*K*_D_) are shown at the top of each sensorgram. **Figure S11.** DNA-protein interaction of Form A. (A) Enlarged view of the polymerase active site. The 3′-terminus of the DNA and the active site residues D965 and D967 in DP2 are colored gold. The positions of the side chains are those of crystal structures and should be regarded as approximate ones. (B) Side view cross section of Form A. The red colored DNA strand is obtained by extending the upstream of the DNA duplex of the *P*. *abyssi* PolD-DNA structure fitted to our Form A map. Note that the DNA orientation of the reported *P*. *abyssi* structure cause a collision with the inner wall of the PCNA channel. (C) The bottom view of atomic model shown in (B). **Figure S12.** Crystal structure of FEN1–PCNA (PDB: 1UL1), superimposed on the EM maps. (A) FEN1 in the X configuration (purple), placed on the free PCNA subunit of Form A map. (B-D) FEN1 (blue) in the X (B), Y (C), and Z (D) configurations placed on the free PCNA subunit of Form B map. Note that only one configuration of FEN1 molecule is displayed in each figure for clarity. (PDF 13043 kb)

## Data Availability

All data generated or analyzed during this study are included in this manuscript and its supplementary information file (Additional file). The obtained structures are deposited in the EMData Bank (accession code: EMD-0723 (Form A) and EMD-0725 (Form B)) and Protein Data Bank (accession code: 6KNB (Form A) and 6KNC (Form B)).

## References

[1] Braithwaite DK, Ito J (1993). Compilation, alignment, and phylogenetic relationships of DNA polymerases. Nucleic Acids Res.

[2] Cann IK, Ishino Y (1999). Archaeal DNA replication: identifying the pieces to solve a puzzle. Genetics.

[3] Lipps G, Rother S, Hart C, Krauss G (2003). A novel type of replicative enzyme harbouring ATPase, primase and DNA polymerase activity. EMBO J.

[4] Ohmori H, Friedberg EC, Fuchs RP, Goodman MF, Hanaoka F, Hinkle D, Kunkel TA, Lawrence CW, Livneh Z, Nohmi T (2001). The Y-family of DNA polymerases. Mol Cell.

[5] Uemori T, Ishino Y, Toh H, Asada K, Kato I (1993). Organization and nucleotide sequence of the DNA polymerase gene from the archaeon Pyrococcus furiosus. Nucleic Acids Res.

[6] Takagi M, Nishioka M, Kakihara H, Kitabayashi M, Inoue H, Kawakami B, Oka M, Imanaka T (1997). Characterization of DNA polymerase from Pyrococcus sp. strain KOD1 and its application to PCR. Appl Environ Microbiol.

[7] Ishino S, Ishino Y (2014). DNA polymerases as useful reagents for biotechnology − the history of developmental research in the field. Front Microbiol.

[8] Uemori T, Sato Y, Kato I, Doi H, Ishino Y (1997). A novel DNA polymerase in the hyperthermophilic archaeon, Pyrococcus furiosus: gene cloning, expression, and characterization. Genes Cells.

[9] Cann IK, Komori K, Toh H, Kanai S, Ishino Y (1998). A heterodimeric DNA polymerase: evidence that members of Euryarchaeota possess a distinct DNA polymerase. Proc Natl Acad Sci U S A.

[10] Forterre P, Elie C, Kohiyama M (1984). Aphidicolin inhibits growth and DNA synthesis in halophilic arachaebacteria. J Bacteriol.

[11] Ishino Y, Ishino S (2001). DNA polymerases from euryarchaeota. Methods Enzymol.

[12] Henneke G, Flament D, Hubscher U, Querellou J, Raffin JP (2005). The hyperthermophilic euryarchaeota Pyrococcus abyssi likely requires the two DNA polymerases D and B for DNA replication. J Mol Biol.

[13] Ishino S, Ishino Y (2006). Comprehensive search for DNA polymerase in the hyperthermophilic archaeon, Pyrococcus furiosus. Nucleosides Nucleotides Nucleic Acids.

[14] Berquist BR, DasSarma P, DasSarma S (2007). Essential and non-essential DNA replication genes in the model halophilic Archaeon, Halobacterium sp. NRC-1. BMC Genet.

[15] Sarmiento F, Mrazek J, Whitman WB (2013). Genome-scale analysis of gene function in the hydrogenotrophic methanogenic archaeon Methanococcus maripaludis. Proc Natl Acad Sci U S A.

[16] Cubonova L, Richardson T, Burkhart BW, Kelman Z, Connolly BA, Reeve JN, Santangelo TJ (2013). Archaeal DNA polymerase D but not DNA polymerase B is required for genome replication in Thermococcus kodakarensis. J Bacteriol.

[17] Sauguet L, Raia P, Henneke G, Delarue M (2016). Shared active site architecture between archaeal PolD and multi-subunit RNA polymerases revealed by X-ray crystallography. Nat Commun.

[18] Aravind L, Koonin EV (1998). Phosphoesterase domains associated with DNA polymerases of diverse origins. Nucleic Acids Res.

[19] Makiniemi M, Pospiech H, Kilpelainen S, Jokela M, Vihinen M, Syvaoja JE (1999). A novel family of DNA-polymerase-associated B subunits. Trends Biochem Sci.

[20] Takashima N, Ishino S, Oki K, Takafuji M, Yamagami T, Matsuo R, Mayanagi K, Ishino Y (2019). Elucidating functions of DP1 and DP2 subunits from the Thermococcus kodakarensis family D DNA polymerase. Extremophiles..

[21] Raia P, Carroni M, Henry E, Pehau-Arnaudet G, Brule S, Beguin P, Henneke G, Lindahl E, Delarue M, Sauguet L (2019). Structure of the DP1-DP2 PolD complex bound with DNA and its implications for the evolutionary history of DNA and RNA polymerases. PLoS Biol.

[22] Tori K, Kimizu M, Ishino S, Ishino Y (2007). DNA polymerases BI and D from the hyperthermophilic archaeon Pyrococcus furiosus both bind to proliferating cell nuclear antigen with their C-terminal PIP-box motifs. J Bacteriol.

[23] Kuba Y, Ishino S, Yamagami T, Tokuhara M, Kanai T, Fujikane R, Daiyasu H, Atomi H, Ishino Y (2012). Comparative analyses of the two proliferating cell nuclear antigens from the hyperthermophilic archaeon, Thermococcus kodakarensis. Genes Cells.

[24] Miyata T, Suzuki H, Oyama T, Mayanagi K, Ishino Y, Morikawa K (2005). Open clamp structure in the clamp-loading complex visualized by electron microscopic image analysis. Proc Natl Acad Sci U S A.

[25] Mayanagi K, Kiyonari S, Saito M, Shirai T, Ishino Y, Morikawa K (2009). Mechanism of replication machinery assembly as revealed by the DNA ligase-PCNA-DNA complex architecture. Proc Natl Acad Sci U S A.

[26] Nishida H, Mayanagi K, Kiyonari S, Sato Y, Oyama T, Ishino Y, Morikawa K (2009). Structural determinant for switching between the polymerase and exonuclease modes in the PCNA-replicative DNA polymerase complex. Proc Natl Acad Sci U S A.

[27] Mayanagi K, Kiyonari S, Nishida H, Saito M, Kohda D, Ishino Y, Shirai T, Morikawa K (2011). Architecture of the DNA polymerase B-proliferating cell nuclear antigen (PCNA)-DNA ternary complex. Proc Natl Acad Sci U S A.

[28] Mayanagi K, Ishino S, Shirai T, Oyama T, Kiyonari S, Kohda D, Morikawa K, Ishino Y (2018). Direct visualization of DNA baton pass between replication factors bound to PCNA. Sci Rep.

[29] Fernandez-Leiro R, Conrad J, Scheres SH, Lamers MH (2015). cryo-EM structures of the *E*. *coli* replicative DNA polymerase reveal its dynamic interactions with the DNA sliding clamp, exonuclease and τ. Elife.

[30] Fernandez-Leiro R, Conrad J, Yang JC, Freund SM, Scheres SH, Lamers MH (2017). Self-correcting mismatches during high-fidelity DNA replication. Nat Struct Mol Biol.

[31] Shen Y, Tang XF, Matsui I (2003). Subunit interaction and regulation of activity through terminal domains of the family D DNA polymerase from Pyrococcus horikoshii. J Biol Chem.

[32] Shen Y, Tang XF, Yokoyama H, Matsui E, Matsui I (2004). A 21-amino acid peptide from the cysteine cluster II of the family D DNA polymerase from Pyrococcus horikoshii stimulates its nuclease activity which is Mre11-like and prefers manganese ion as the cofactor. Nuc Acids Res.

[33] Suwa Y, Gu J, Baranovskiy AG, Babayeva ND, Pavlov YI, Tahirov TH (2015). Crystal structure of the human Pol α B subunit in complex with the C-terminal domain of the catalytic subunit. J Biol Chem.

[34] Baranovskiy AG, Gu J, Babayeva ND, Kurinov I, Pavlov YI, Tahirov TH (2017). Crystal structure of the human Polε B-subunit in complex with the C-terminal domain of the catalytic subunit. J Biol Chem.

[35] Matsumiya S, Ishino S, Ishino Y, Morikawa K (2002). Physical interaction between proliferating cell nuclear antigen and replication factor C from *Pyrococcus furiosus*. Genes Cells.

[36] Crooks GE, Hon G, Chandonia JM, Brenner SE (2004). WebLogo: a sequence logo generator. Genome Res.

[37] Gulbis JM, Kelman Z, Hurwitz J, O'Donnell M, Kuriyan J (1996). Structure of the C-terminal region of p21(WAF1/CIP1) complexed with human PCNA. Cell..

[38] Sakurai S, Kitano K, Yamaguchi H, Hamada K, Okada K, Fukuda K, Uchida M, Ohtsuka E, Morioka H, Hakoshima T (2005). Structural basis for recruitment of human flap endonuclease 1 to PCNA. EMBO J.

[39] Greenough L, Kelman Z, Gardner AF (2015). The roles of family B and D DNA polymerases in Thermococcus species 9°N Okazaki fragment maturation. J Biol Chem.

[40] Madru C, Henneke G, Raia P, Hugonneau-Beaufet I, Pehau-Arnaudet G, England Lindahl E, Delarue M, Carroni M (2020). Sauguet L Structural basis for the increased processivity of D-family DNA polymerases in complex with PCNA. Nat Commun..

[41] Castrec B, Rouillon C, Henneke G, Flament D, Querellou J, Raffin JP (2009). Binding to PCNA in euryarchaeal DNA replication requires two PIP motifs for DNA polymerase D and one PIP motif for DNA polymerase B. J Mol Biol.

[42] Lancey C, Tehseen M, Raducanu VS, Rashid F, Merino N, Ragan TJ, Savva CG, Zaher MS, Shirbini A, Blanco FJ, Hamdan SM, De Biasio A (2020). Structure of the processive human Pol δ holoenzyme. Nat Commun.

[43] Zheng SQ, Palovcak E, Armache JP, Verba KA, Cheng Y, Agard DA (2017). MotionCor2: anisotropic correction of beam-induced motion for improved cryo-electron microscopy. Nat Methods.

[44] Rohou A, Grigorieff N (2015). CTFFIND4: fast and accurate defocus estimation from electron micrographs. J Struct Biol.

[45] Kimanius D, Forsberg BO, Scheres SH, Lindahl E (2016). Accelerated cryo-EM structure determination with parallelisation using GPUs in RELION-2. Elife.

[46] Ladner JE, Pan M, Hurwitz J, Kelman Z (2011). Crystal structures of two active proliferating cell nuclear antigens (PCNAs) encoded by Thermococcus kodakaraensis. Proc Natl Acad Sci U S A.

[47] Bubeck D, Reijns MA, Graham SC, Astell KR, Jones EY, Jackson AP (2011). PCNA directs type 2 RNase H activity on DNA replication and repair substrates. Nucleic Acids Res.

[48] Sebesta M, Cooper CDO, Ariza A, Carnie CJ, Ahel D (2017). Structural insights into the function of ZRANB3 in replication stress response. Nat Commun.

[49] Webb B, Sali A (2014). Comparative protein structure modeling using MODELLER. Curr Protoc Bioinformatics.

[50] Adams PD, Afonine PV, Bunkoczi G, Chen VB, Davis IW, Echols N, Headd JJ, Hung LW, Kapral GJ, Grosse-Kunstleve RW (2010). PHENIX: a comprehensive Python-based system for macromolecular structure solution. Acta Crystallogr D Biol Crystallogr.

[51] Afonine PV, Poon BK, Read RJ, Sobolev OV, Terwilliger TC, Urzhumtsev A, Adams PD (2018). Real-space refinement in PHENIX for cryo-EM and crystallography. Acta Crystallogr D Struct Biol.

[52] Emsley P, Lohkamp B, Scott WG, Cowtan K (2010). Features and development of Coot. Acta Crystallogr D Biol Crystallogr.

[53] Chen VB, Arendall WB, Headd JJ, Keedy DA, Immormino RM, Kapral GJ, Murray LW, Richardson JS, Richardson DC (2010). MolProbity: all-atom structure validation for macromolecular crystallography. Acta Crystallogr. D Biol Crystallogr..

[54] Komori K, Ichiyanagi K, Morikawa K, Ishino Y (1999). PI-PfuI and PI-PfuII, intein-coded homing endonucleases from Pyrococcus furiosus. II. Characterization of the binding and cleavage abilities by site-directed mutagenesis. Nucleic Acids Res.

[55] Tahirov TH, Makarova KS, Rogozin IB, Pavlov YI, Koonin EV (2009). Evolution of DNA polymerases: an inactivated polymerase-exonuclease module in Pol epsilon and a chimeric origin of eukaryotic polymerases from two classes of archaeal ancestors. Biol Direct.

