# Supplementary material for: Two conformations of DNA polymerase D-PCNA-DNA, an archaeal replisome complex, revealed by cryo-electron microscopy
Source: BMC Biol. 2020 Oct 28;18:152. doi: 10.1186/s12915-020-00889-y (PMC7594292; doi:10.1186/s12915-020-00889-y)
Supplement: Supplementary file 1 — Table S1. Model refinement statistics. Figure S1. The synthetic deoxyoligonucleotides used in this study. Bases with lower case letters are linked by phosphorothioate bond for protection from the 3′–5′ exonuclease activity. Figure S2. Sample preparations. (A) Gel filtration chromatography of the reconstituted PolD-PCNA-DNA (30/45) complex. The absorbances at 260 nm and 280 nm are indicated by red and blue lines, respectively. (B) The peak fractions in (A) were analyzed by a gradient (10–20%) SDS-PAGE. The lanes for the purified proteins used for complex reconstitution and the markers are indicated by the notations “PCNA,” “PolD,” and “Marker,” respectively. (C) Gel filtration chromatography of the reconstituted PolD-PCNA-DNA (25/35) complex. (D) The peak fraction indicated by the asterisk (*) in (C) was analyzed by a gradient (10–20%) SDS-PAGE. Figure S3. Electron microscopic images of PolD-PCNA-DNA complex. (A) Representative electron microscopic image of negatively stained PolD-PCNA-DNA (30/45) complex, recorded by a CCD camera. (B) Representative cryo-electron microscopic image of PolD-PCNA-DNA complex, recorded by a CCD camera, without Volta Phase Plate (VPP). The white and magenta broken circles indicate tetrameric and pentameric clusters of the complex, respectively. (C) Representative cryo-electron microscopic image of PolD-PCNA-DNA complex, recorded by a Falcon II direct electron detector, using VPP. The white and magenta broken circles indicate tetrameric and pentameric clusters of the complex, respectively. (D) Representative cryo-electron microscopic image of PolD-PCNA-DNA complex, recorded by a Falcon 3EC direct electron detector without phase plate. The scale bars indicate 50 nm. Figure S4. Flow charts of 3D classification and refinement procedures for the PolD-PCNA-DNA (30/45) complex. The details are described in the Methods section. Figure S5. Flow charts of 3D classification and refinement procedures for the PolD-PCNA-DNA (25/35) complex. The detail [file 12915_2020_889_MOESM1_ESM.pdf]

Additional Information

Two conformations of DNA polymerase D-PCNA-DNA, an archaeal replisome complex, revealed by cryo-electron microscopy

Kouta Mayanagi[1†*], Keisuke Oki[†2], Naoyuki Miyazaki[3,4], Sonoko Ishino[2], Takeshi Yamagami[2], Kosuke Morikawa[5], Kenji Iwasaki[3,4], Daisuke Kohda[1], Tsuyoshi Shirai[6*], and Yoshizumi Ishino[2*]

Table S1. Model refinement statistics

	Form A (6KNB)		Form B (6KNC)	
Resolution Estimates (Å)	Masked	Unmasked	Masked	Unmasked
d FSC (half maps; 0.143)	7.00	7.30	9.50	10.10
d 99 (full/half1/half2)	7.3/3.3/3.3	7.3/2.9/2.9	9.8/12.7/12.6	9.5/9.3/8.9
d model	6.90	7.00	9.20	9.30
d FSC model (0/0.143/0.5)	5.8/6.6/7.3	6.4/6.8/7.7	8.1/8.8/10.9	8.3/9.0/11.6
Model-Map correlation				
CC (mask)	0.72		0.76	
CC (box)	0.76		0.86	
CC (peaks)	0.62		0.65	
CC (volume)	0.70		0.72	
Mean CC for ligands	0.75		0.79	
Model statistics				
Bonds (RMSD)				
Length (Å)	0.003		0.010	
Angles (°)	0.90		0.99	
MolProbity score	1.95		1.70	
Clash score	12.63		12.67	
Ramachandran plot (%)				
Outliers	0.00		0.00	
Allowed	4.92		2.40	
Favored	95.08		97.60	
Rotamer outliers (%)	0.65		0.20	
Cβ outliers (%)	0.00		0.00	
Peptide plane (%)				
Cis proline/general	4.5/0.0		4.5/0.0	
Twisted proline/general	0.0/0.0		0.0/0.0	
CaBLAM outliers (%)	3.48		2.77	

temp45EMssss:
5′-TGAGGTGATCGTTCGCTACATGTCGTCAGGATTCCAGGCAgttcG

pri30EMssss:
5′-CGAACTGCCTGGAATCCTGACGACAtgtaG

temp35EMssss:
5′-TGAGGTGATCGTTCGCTACATGTCGTCAGGATTCCAGGCAgttcG

pri25EMssss:
5′-CGAACTGCCTGGAATCCTGAcgacA

5′Cy5 pri32:
Cy5-5′-CGAACTGCCTGGAATCCTGACGACATGTAGCG

temp45:
5′-TGAGGTGATCGTTCGCTACATGTCGTCAGGATTCCAGGCAGTTCG

Figure S1. The synthetic deoxyoligonucleotides used in this study. Bases with lower case letters are linked by phosphorothioate bond for protection from the 3′–5′ exonuclease activity.

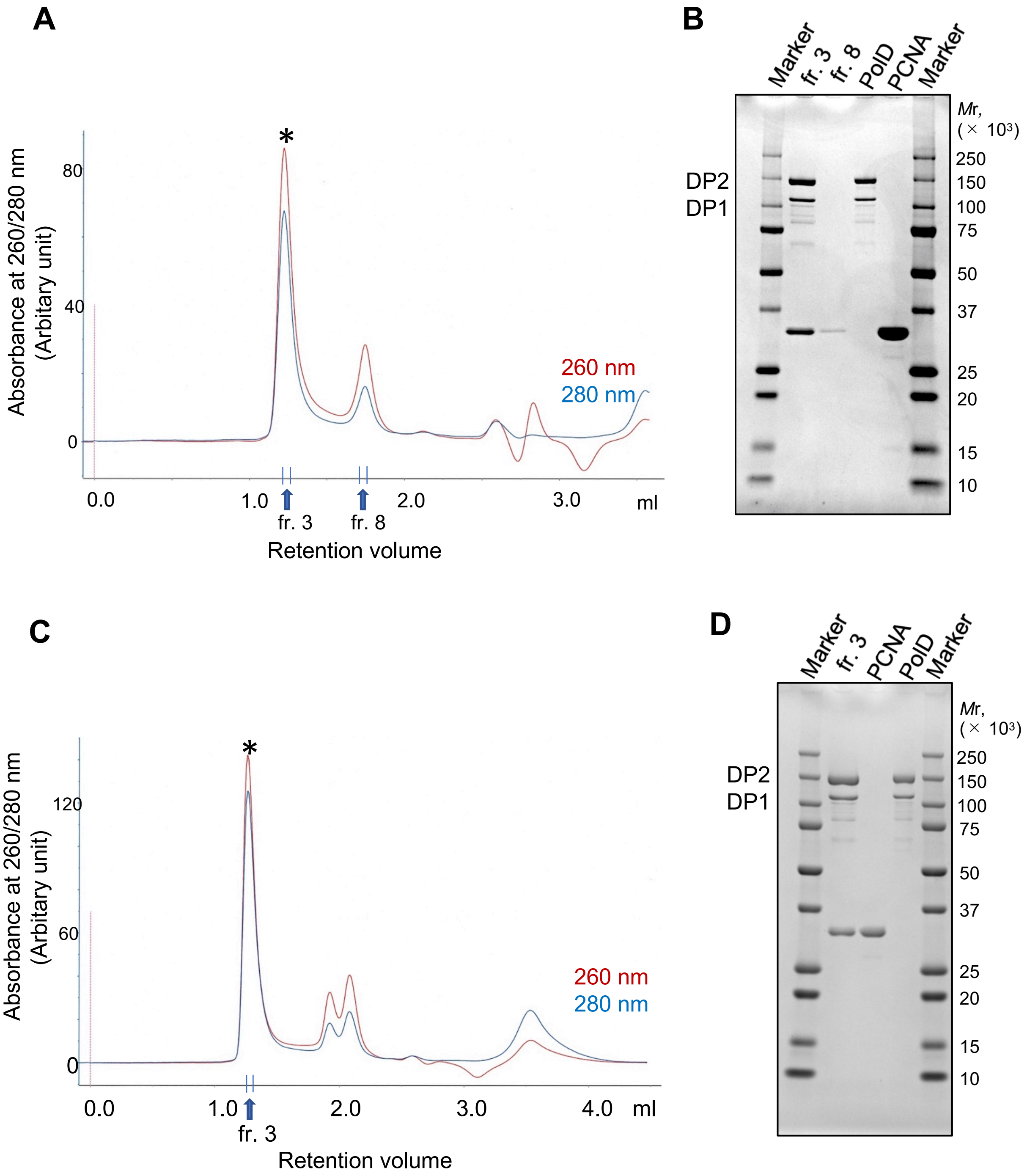

Figure S2. Sample preparations. (A) Gel filtration chromatography of the reconstituted PolD-PCNA-DNA (30/45) complex. The absorbances at 260 nm and 280 nm are indicated by red and blue lines, respectively. (B) The peak fractions in (A) were analyzed by a gradient (10-20%) SDS-PAGE. The lanes for the purified proteins used for complex reconstitution and the markers are indicated by the notations “PCNA,” “PolD,” and “Marker,” respectively. (C) Gel filtration chromatography of the reconstituted PolD-PCNA-DNA (25/35) complex. (D) The peak fraction indicated by the asterisk (*) in (C) was analyzed by a gradient (10-20%) SDS-PAGE.

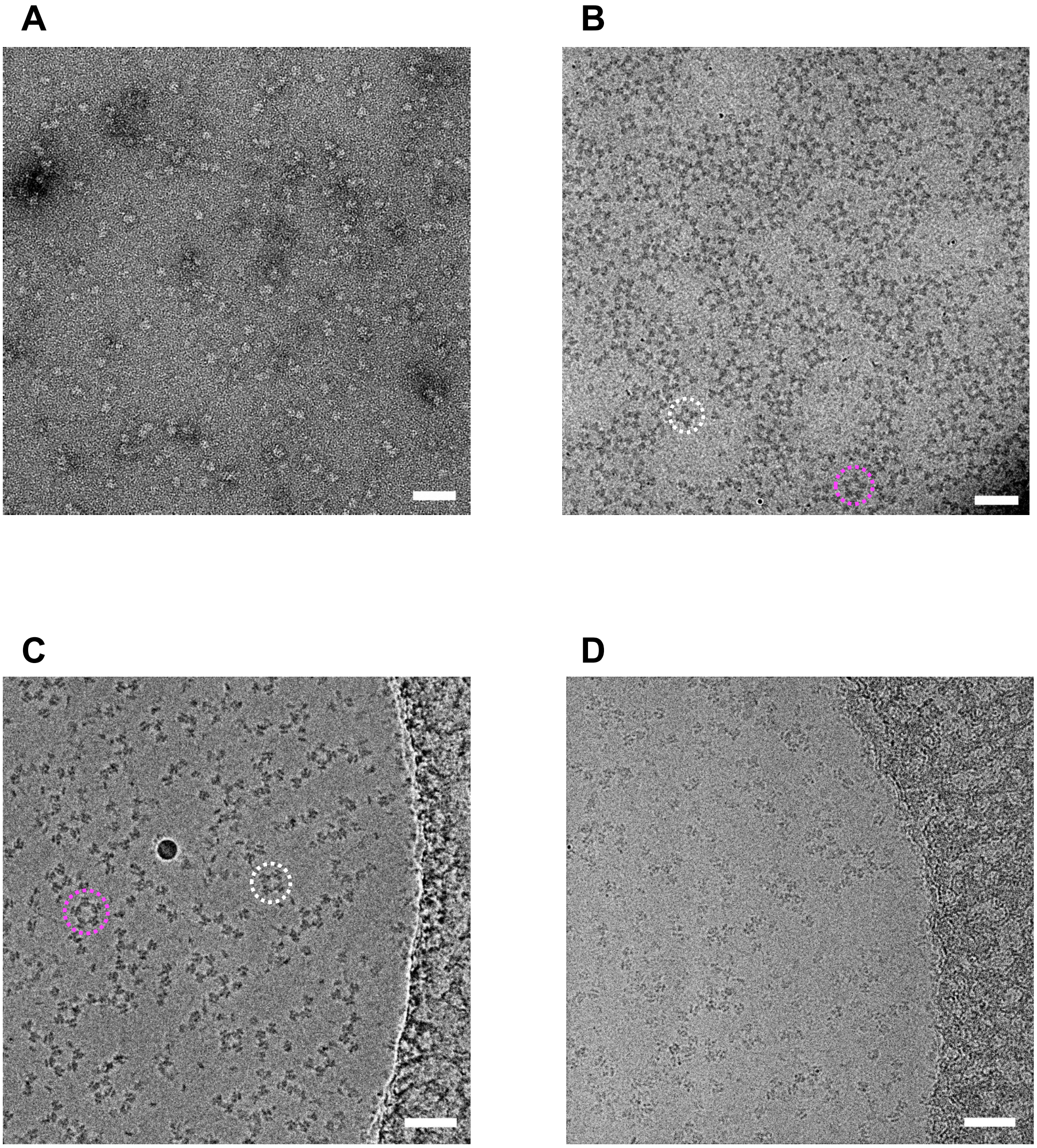

Figure S3. Electron microscopic images of PolD-PCNA-DNA complex. (A) Representative electron microscopic image of negatively stained PolD-PCNA-DNA (30/45) complex, recorded by a CCD camera. (B) Representative cryo-electron microscopic image of PolD-PCNA-DNA complex, recorded by a CCD camera, without Volta Phase Plate (VPP). The white and magenta broken circles indicate tetrameric and pentameric clusters of the complex, respectively. (C) Representative cryo-electron microscopic image of PolD-PCNA-DNA complex, recorded by a Falcon II direct electron detector, using VPP. The white and magenta broken circles indicate tetrameric and pentameric clusters of the complex, respectively. (D) Representative cryo-electron microscopic image of PolD-PCNA-DNA complex, recorded by a Falcon 3EC direct electron detector without phase plate. The scale bars indicate 50 nm.

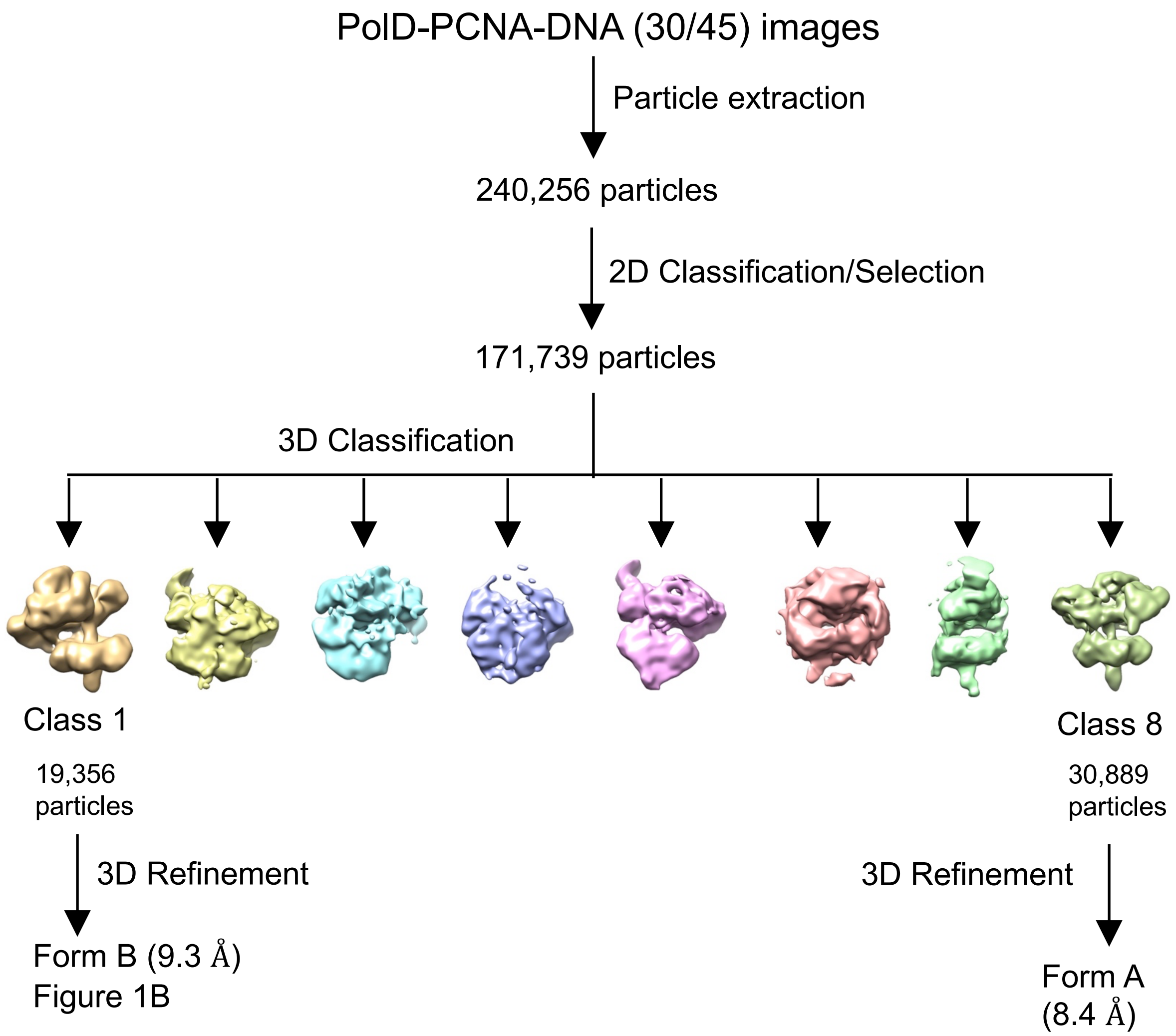

Figure S4. Flow charts of 3D classification and refinement procedures for the PolD-PCNA-DNA (30/45) complex. The details are described in the Methods section.

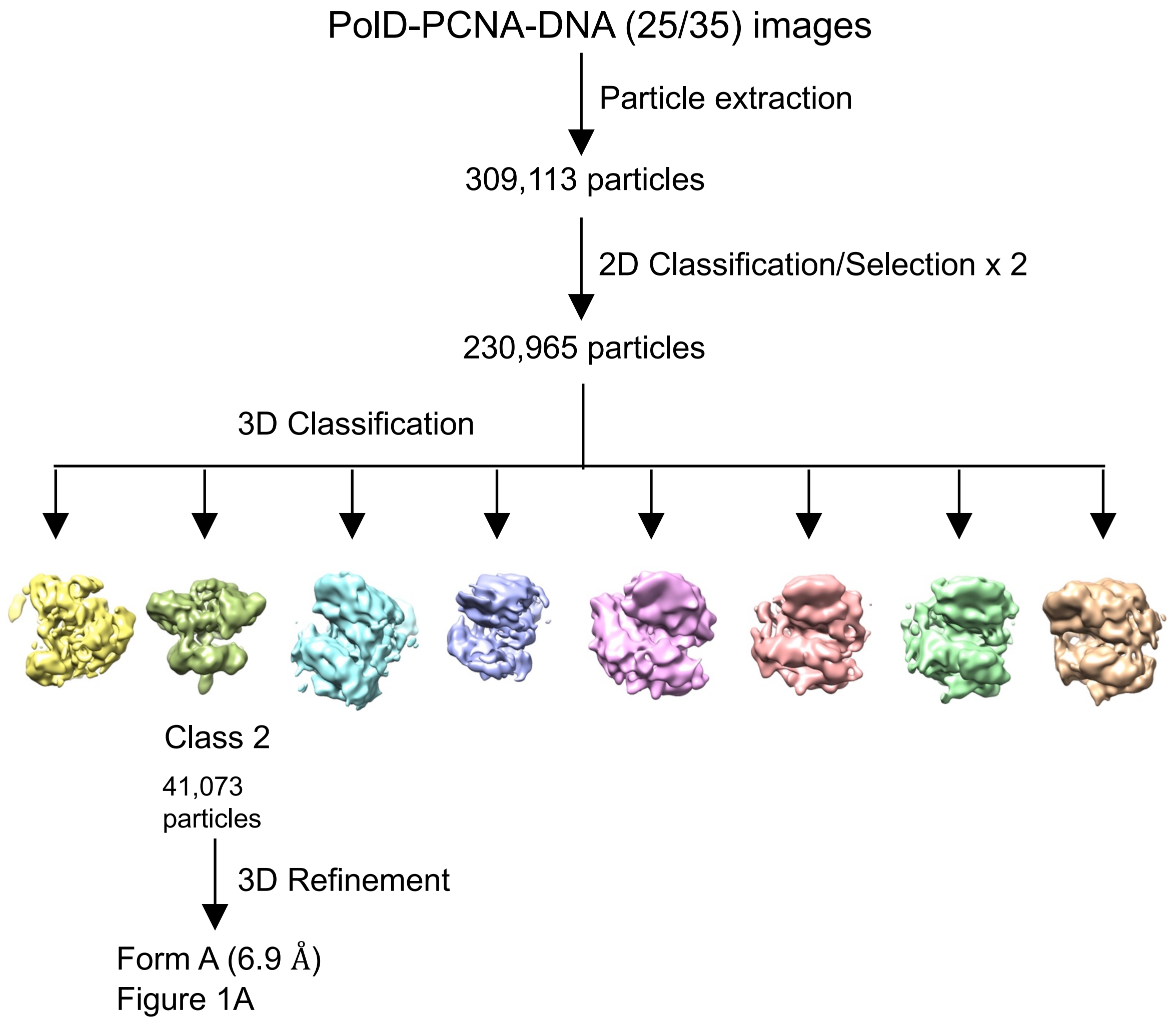

Figure S5. Flow charts of 3D classification and refinement procedures for the PolD-PCNA-DNA (25/35) complex. The details are described in the Methods section.

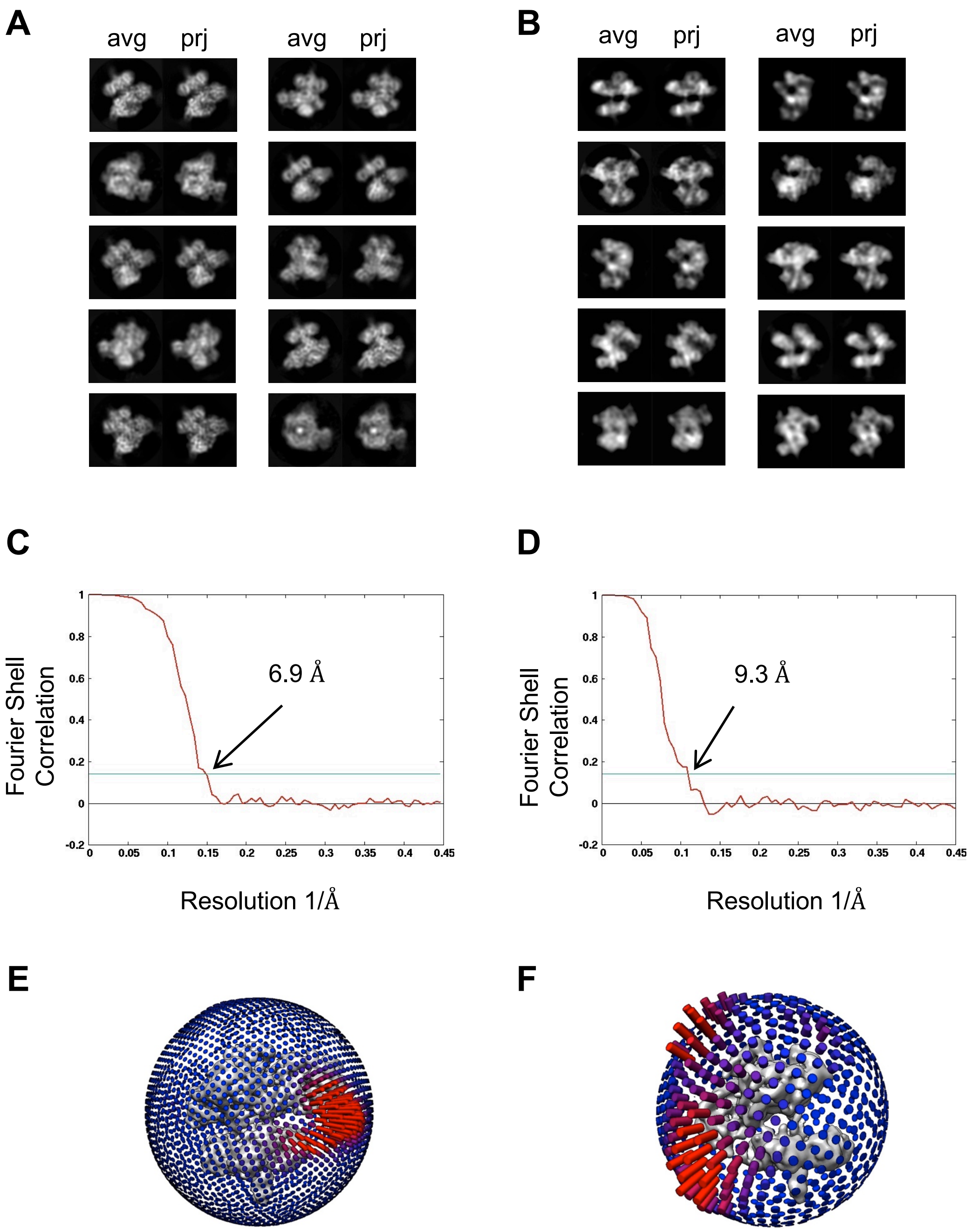

Figure S6. EM analysis. (A) Comparison between representative 2D class averages (avg) and projections from the 3D model (prj) of the Form A complex. (B) Comparison between representative 2D class averages (avg) and projections from the 3D model (prj) of the Form B complex. Gold standard Fourier shell correction (FSC) curves for the EM density maps of the form A complex (C), and Form B complex (D). Resolutions are given for the FSC 0.143 criteria. Euler angle distributions of particles used for the final maps of, (E) the Form A complex, and (F) the Form B complex.

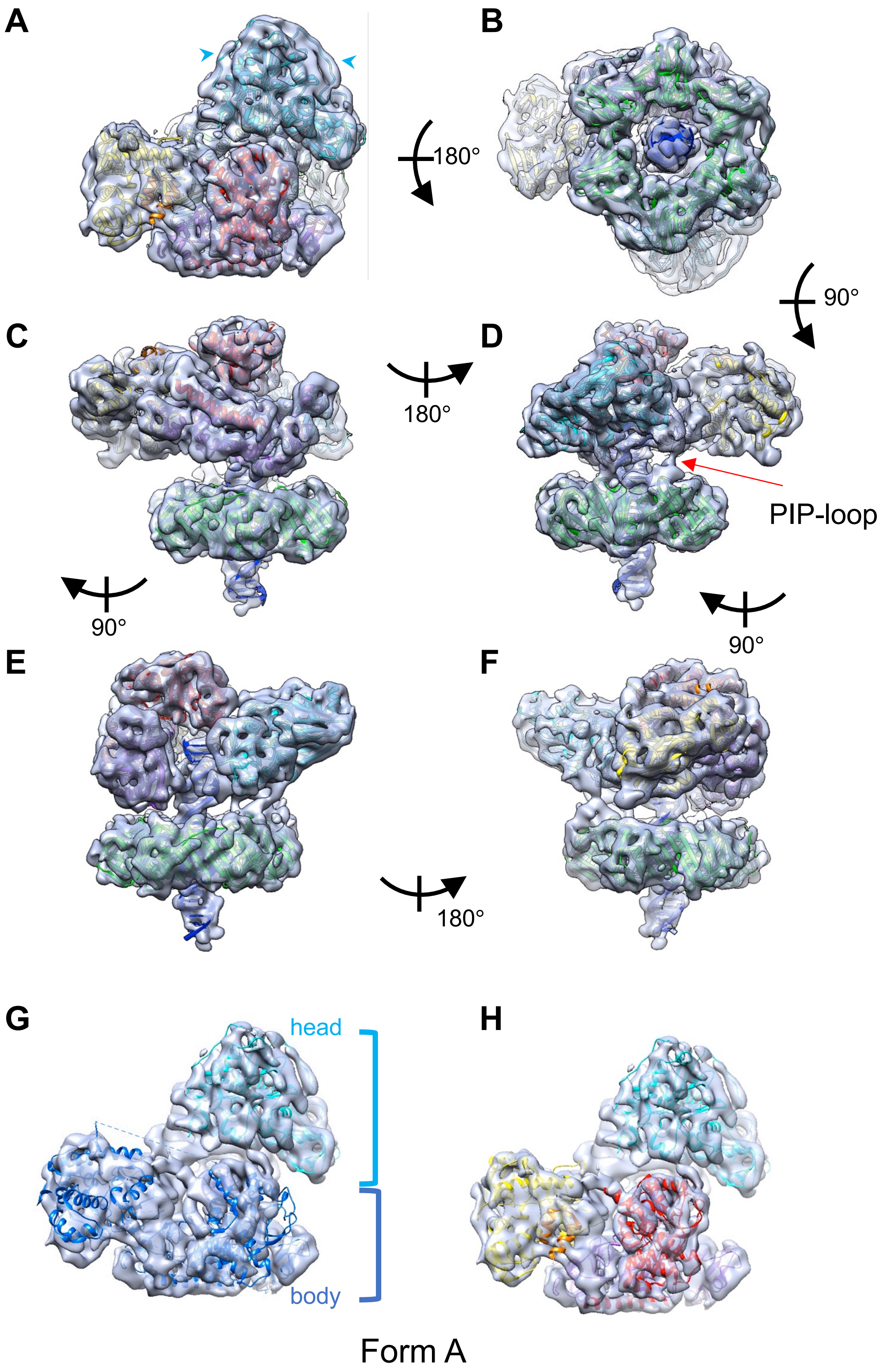

Figure S7. 3D map and atomic model fitting of PolD-PCNA-DNA complex (Form A). (A) Top view. The arrows with the angles indicate rotations to see the bottom view (B) and the side views of (C), (D), and (E), respectively. The PCNA-interacting C-terminal loop of DP2, harboring the PIP-box, is indicated by the red arrow in (D). The crystal structures of *T. kodakarensis* PCNA and *P. abyssi* DP1 are shown in green and cyan ribbon. The N-terminal, catalytic, center, and C-terminal domains of *P. abyssi* DP2 are shown in red, purple, yellow, and orange ribbon, respectively. (G) Top view of the Form A map, with the crystal structures of DP1 ((PDB: 5IHE, cyan) and DP2 (PDB: 5IJL, blue) docked as rigid bodies. (H) Top view of the Form A map in which each domain of DP2 (N-terminal (red), catalytic (purple), center (yellow), and C-terminal (orange)) is docked independently into the map. Note that the PCNA region of the map is omitted for clarity.

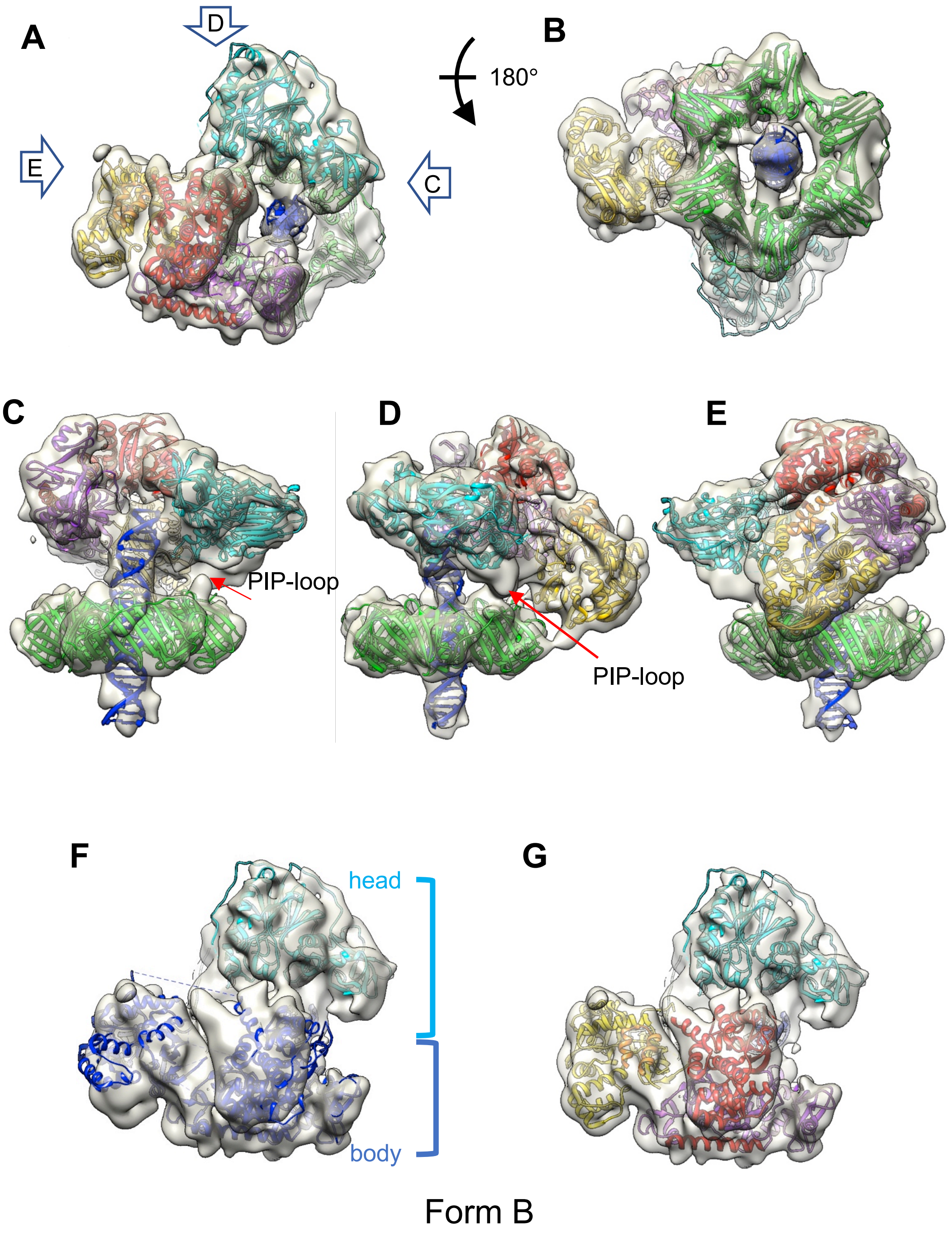

Figure S8. 3D map and atomic model fitting of PolD-PCNA-DNA complex (Form B). (A) Top view. The arrows with the notation of C, D, E indicates the view angle of side views of (C), (D), and (E), respectively. (B) Bottom view. (C) – (E) Side views. The PCNA-interacting C-terminal loop of DP2, harboring the PIP-box, is indicated by the red arrow. The crystal structures of PCNA and DP1 are shown in green and cyan ribbons, respectively. The N-terminal, catalytic, center, and C-terminal domains of DP2 are shown in red, purple, yellow, and orange ribbon, respectively. (F) Oblique view of the Form B map, in which PolD is aligned in the same direction as Supplementary Figure S7G. The crystal structures of DP1 (PDB: 5IHE, cyan) and DP2 (PDB: 5IJL, blue) are docked as rigid bodies into head and body region, respectively. (G) The same view as shown in (F), in which each domain of DP2 (N-terminal (red), catalytic (purple), center (yellow), and C-terminal (orange)) is docked independently into the map. Note that the PCNA region of the map is omitted for clarity.

A

```
                     α1             β1
p261C     2194 ------DSSAIEMTLVEVLQKKLMAFTLQDLVCLK--CRGVKET 2230
p180C     1318 --SPLTFTVQLSNKLIMDIRRFIKKYYDGWLICEEPTCRNRTR- 1358
DP2C      1101 EHH--VAETIINSHLVPDLRGNLRSFTRQEFRCVK--CNTKYR- 1139
consensus             :: :: ::: :::  :  ::   : * :  *::  :

                                   β2       α2
p261C     2231 SMPV-------YCS--CAGDFALTIHTQVFMEQIGIFRNIAQH- 2264
p180C     1359 HLPLQFSRTGPLCPACMKATLQPEYSDKSLYTQLCFYRYIFDAE 1402
DP2C      1140 RPPLTG-----KCPK-CGGKIVLTVSKGAIEKYLPTAKMLVTK- 1176
consensus      : *:       :*:  : :::  :::: :::::::::: :::

                                            α3
p261C     2265 -----------------Y--------GMSYLLETLE-WLLQK  2280
p180C     1403 CALEKLTTDHEKDKLKKQFFTPKVLQDYRKLKNTAEQFL---  1440
DP2C      1177 -----------------YRVKDYTRQRICITEKDIK-TLF--  1198
consensus                       :         : ::::::: :*
```

B

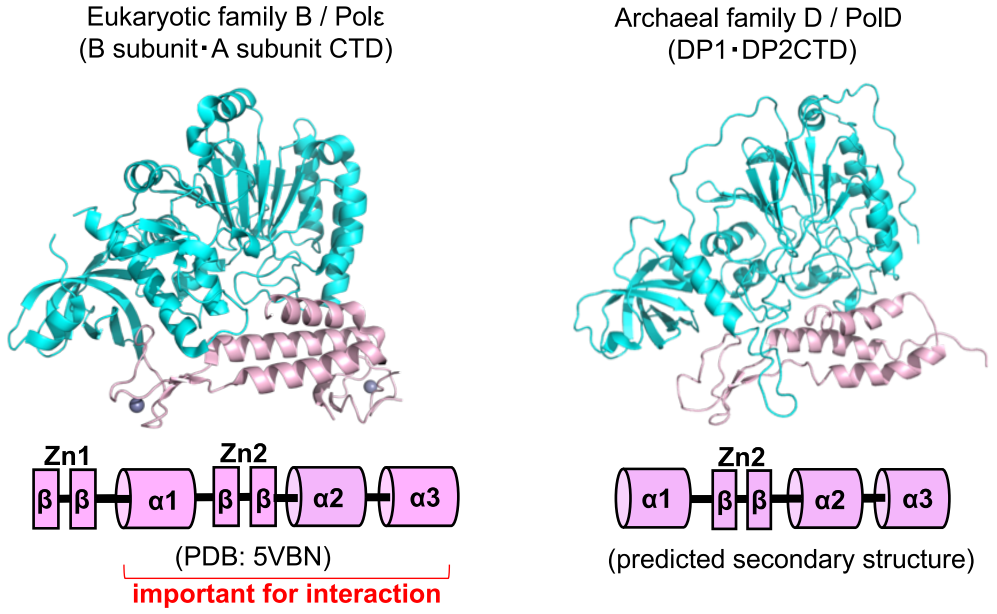

Figure S9. Structural similarity between eukaryotic family B and archaeal family D DNA polymerases. (A) Structure-based sequence alignment of the C-terminal domains of p261 of Polε (p261C), p180 of DP2 (p180C) and DP2 of PolD (DP2C) were performed with reference to a previous report [55]. Amino-acid sequences, belonging to α1–3 residues, are aligned by PROMALS3D (PROfile Multiple Alignment with predicted Local Structures and 3D constraints), and colored as in Figure 3. In the bottom line, “*” and “:” indicate identical and similar amino acid residues, respectively. (B) Crystal structure of human Polε (left, 5VBN), and model structure of *T. kodakarensis* PolD (right). Secondary structures of the C-terminal domains are shown in schematic representations. Zn1, Zn2, Zinc-finger; α1–3, alpha helix; β, beta sheet.

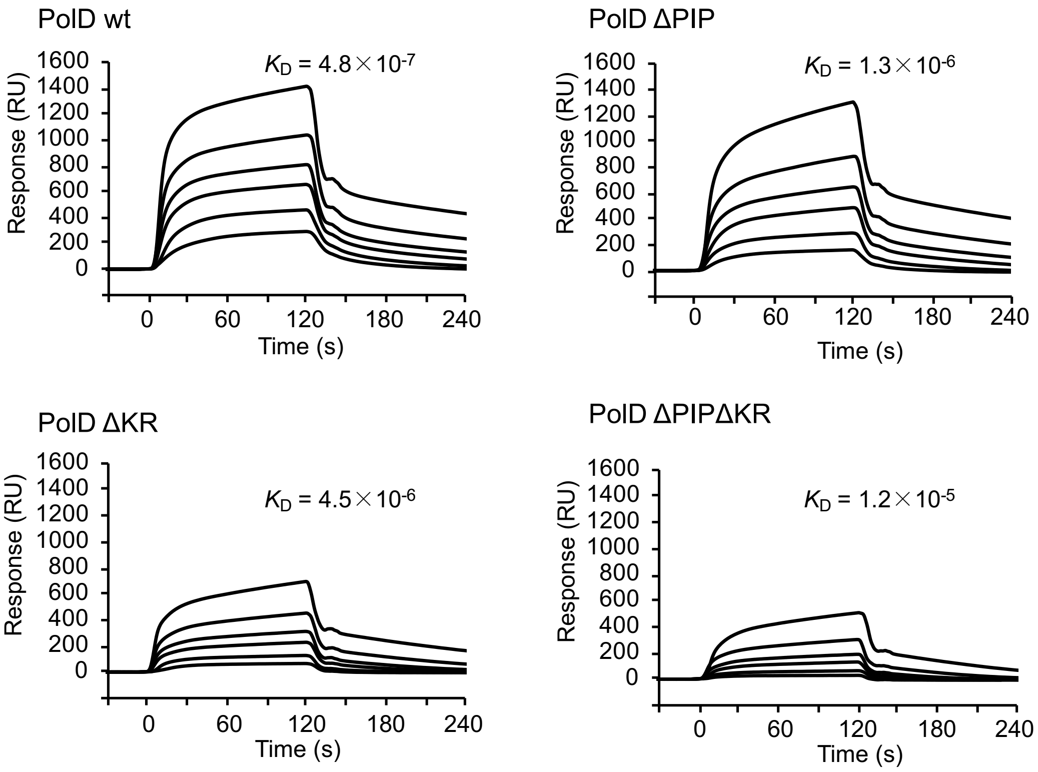

Figure S10. Interactions between PCNA and various PolD mutants. SPR analyses were performed to detect the physical interactions of PCNA with various PolD mutants (wt, ΔPIP, ΔKR, and ΔPIPΔKR). Purified PCNA was immobilized on a sensor chip, and six different concentrations (50, 100, 200, 300, 500, 1000 nM) of purified PolDs were analysed. The apparent equilibrium constants (K_D) are shown at the top of each sensorgram.

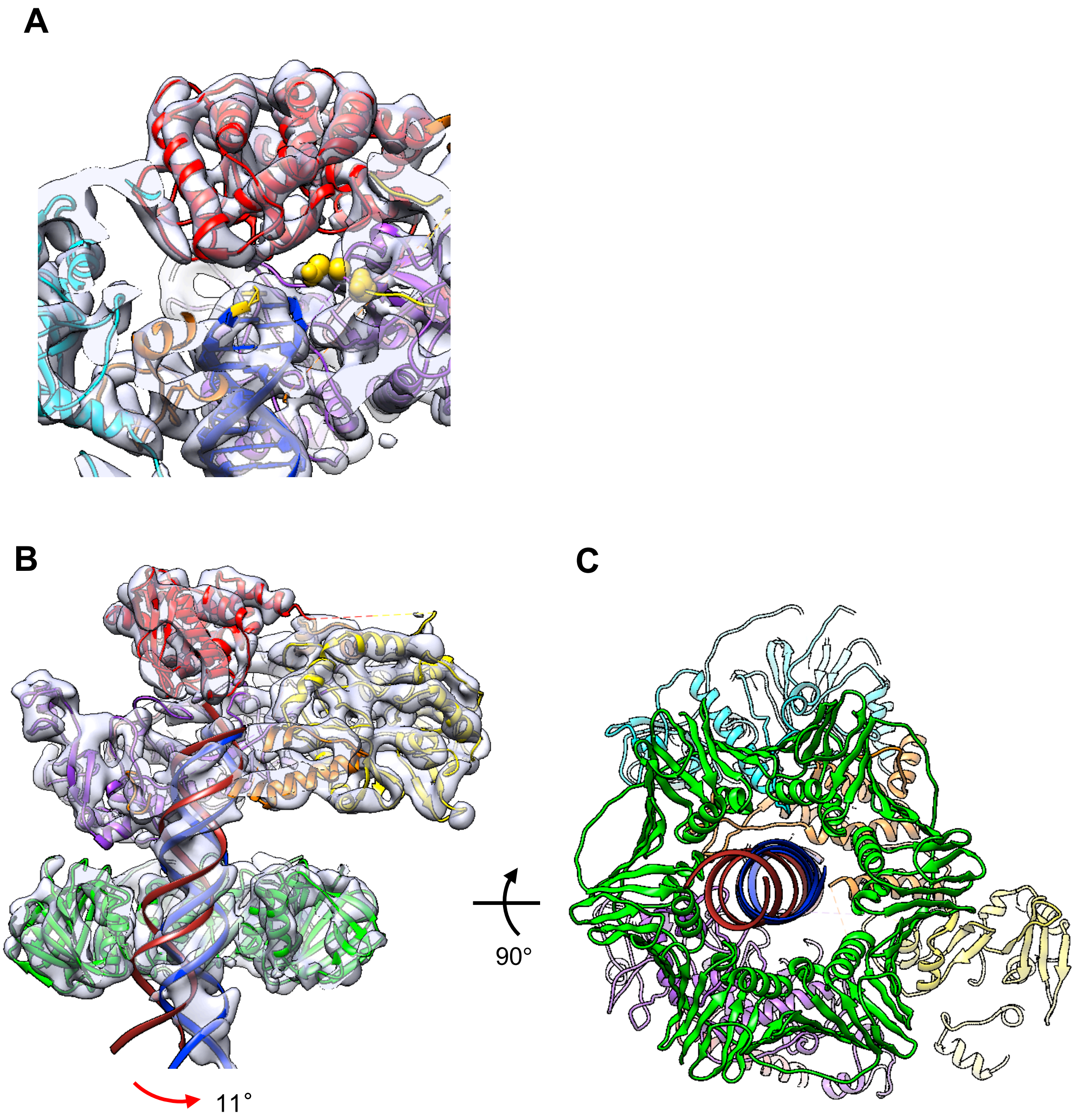

Figure S11. DNA-protein interaction of Form A. (A) Enlarged view of the polymerase active site. The 3′-terminus of the DNA and the active site residues D965 and D967 in DP2 are colored gold. The positions of the side chains are those of crystal structures and should be regarded as approximate ones. (B) Side view cross section of Form A. The red colored DNA strand is obtained by extending the upstream of the DNA duplex of the *P. abyssi* PolD-DNA structure fitted to our form A map. Note that the DNA orientation of the reported *P. abyssi* structure cause a collision with the inner wall of the PCNA channel. (C) The bottom view of atomic model shown in (B).

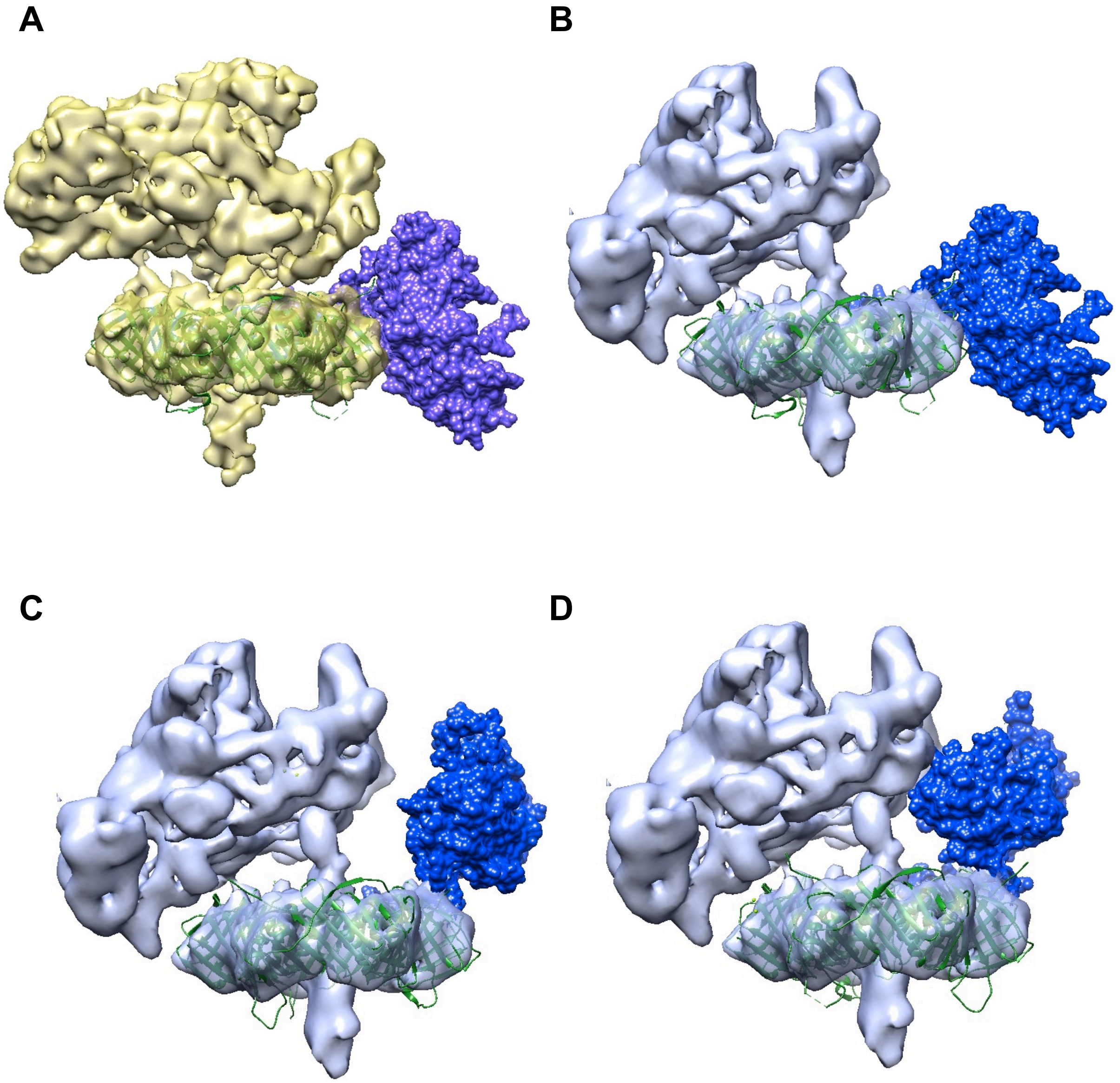

Figure S12. Crystal structure of FEN1–PCNA (PDB: 1UL1), superimposed on the EM maps. (A) FEN1 in the X configuration (purple), placed on the free PCNA subunit of Form A map. (B-D) FEN1 (blue) in the X (B), Y (C), and Z (D) configurations placed on the free PCNA subunit of Form B map. Note that only one configuration of FEN1 molecule is displayed in each figure for clarity.
